# Clinically approved HIF-PHIs modulate redox metabolism, cell growth, and angiogenesis independent of HIF-1α/HIF-2α

**DOI:** 10.1016/j.redox.2026.104206

**Published:** 2026-05-14

**Authors:** Daniela Mennerich, Fawzi Khoder-Agha, Mustafa Beter, Elitsa Y. Dimova, Seppo Ylä-Herttuala, Thomas Kietzmann

**Affiliations:** a Faculty of Biochemistry and Molecular Medicine, and Biocenter Oulu, University of Oulu, FI-90014, Finland; b A.I. Virtanen Institute for Molecular Sciences, University of Eastern Finland, Kuopio, FI-70211, Finland

**Keywords:** HIF-1α, HIF-2α, Roxadustat, Molidustat, RNAseq

## Abstract

HIF-prolyl hydroxylase inhibitors are used to treat anemia in chronic kidney disease. These drugs stabilize hypoxia-inducible factors HIF-1α and HIF-2α, which activate erythropoiesis and iron metabolism pathways. Clinically approved HIF-PHIs including roxadustat and molidustat exhibit distinct molecular structures and selectivity profiles, yet their HIF-independent effects remain poorly understood. Here we show that roxadustat and molidustat modulate mitochondrial function, oxidative stress, lysosomal activity, and lipid accumulation, resulting in distinct cellular phenotypes in HIF-null cells. Notably, roxadustat exhibited anti-proliferative and anti-angiogenic activity in HIF-null cells, contradicting expectations of VEGF-driven angiogenesis via HIF stabilization. RNA sequencing and pathway analysis revealed compound-specific off-target gene regulation affecting cellular processes beyond canonical hypoxia responses including energy metabolism and immune signaling. These findings illuminate mechanisms underlying potential adverse effects-such as thrombosis- and identify alternative therapeutic pathways, providing a framework for optimizing HIF–PHI safety profiles and expanding their clinical applications in oncology and metabolic disorders.

## Graphical abstract

**Figure:**
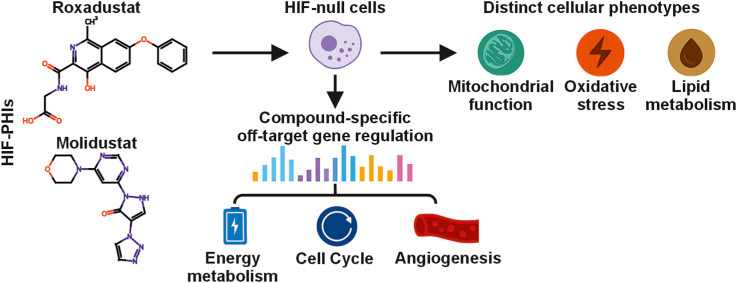

## 1 Introduction

Anemia is one of the most common complications in patients with chronic kidney disease (CKD), primarily caused by reduced erythropoietin (EPO) production and impaired iron metabolism [1]. Traditional treatments rely on erythropoiesis-stimulating agents (ESAs) and iron supplementation. However, a novel therapeutic class, HIF-prolyl hydroxylase inhibitors (HIF-PHIs), has emerged as an alternative approach with several advantages. Unlike traditional ESAs, HIF-PHIs elevate endogenous EPO within a more physiological range [2] while coordinately upregulating iron metabolism proteins (CYBRD1, SLC11A2, SLC40A1) improving iron utilization in patients requiring supplementation.

Five HIF-PHIs are currently approved for clinical use in CKD-related anemia: Roxadustat (FG-4592, FibroGen) was the first approved, with regulatory authorization in China, Japan, the EU, and the UK [3]. Daprodustat (GSK1278863, GlaxoSmithKline) and vadadustat (AKB-6548, Akebia) were approved in 2020 in Japan [4] with vadadustat subsequently gaining approval in the EU and US [5]. The most recent approvals, in 2022, are enarodustat (JTZ-951, Japan Tobacco) in Japan, China, and South Korea [6], and molidustat (BAY85-3934, Bayer) in Japan [7].

HIF-PHIs exert their therapeutic effects by pharmacologically mimicking hypoxia via inhibition of hypoxia-inducible factor (HIF) prolyl hydroxylase domain enzymes (PHD1/*EGLN2*, PHD2/*EGLN1*, PHD3/*EGLN3*). These enzymes serve as cellular oxygen sensors and require molecular oxygen (O_2_), ferrous iron (Fe^2+^), 2-oxoglutarate (2-OG), and ascorbate for their action [8,9]. HIFs are heterodimeric transcription factors composed of a labile α-subunit (HIF-α) and a constitutively expressed β-subunit (HIF-β; also known as ARNT) [10,11]. So far, three α-subunit isoforms: HIF-1α, HIF-2α, and HIF-3α have been identified [10,[12], [13], [14]]. Among these, HIF-2α functions as the primary transcriptional regulator of renal EPO synthesis [15] while dysregulation of the others is more associated with metabolic disorders, and cancer [16,17].

Under normoxia, PHDs catalyze the hydroxylation of specific proline residues on HIF-α subunits [8,18]. This hydroxylation triggers von Hippel-Lindau protein (pVHL)-mediated ubiquitination and proteasomal HIF α-subunit degradation [19]. Among the three PHD isoforms, PHD2 is the most critical regulator, while PHD1 and PHD3 contribute in specific cellular contexts [20].

During hypoxia, the activity of PHDs is inhibited due to insufficient oxygen levels. As a result, stabilized HIF α subunits move to the nucleus, where they dimerize with HIF-β subunits to activate the transcription of more than 300 target genes that orchestrate cellular adaptation to low oxygen, including erythropoiesis via EPO [21,22], angiogenesis via VEGF [23], and glycolytic reprogramming via enzymes such as PGK1, HK2, and LDHA [24,25].

Initial attempts to inhibit PHDs focused on mimicking 2-OG binding, as achieved with N-oxalylglycine (NOG) and its cell-permeable prodrug dimethyloxalylglycine (DMOG) [26]. Both compounds bind the active Fe^2+^ similarly to the 2-OG oxalyl group and contain a glycineamide side chain that occupies the same binding pocket as the methylene groups and C5 carboxylate of 2-OG [27,28]. While effective, these compounds lack isoform selectivity, inhibiting all three PHD isoforms and other members of the approximately 70 human 2-OG-dependent oxygenases family [27].

Roxadustat, a pyridine carboxamide derivative, also inhibits all three PHD isoforms and additionally targets collagen prolyl-4-hydroxylases (CP4H), leading to off-target consequences such as dysregulated connective tissue remodeling and blood pressure alterations [29]. Clinically, it has also been associated with stronger suppression of hepcidin and enhanced iron mobilization. Moreover, roxadustat-mediated HIF-1α activation can modulate iron metabolism and dopamine transporter function in the brain, suggesting broader biological effects beyond erythropoiesis [29,30].

In contrast, molidustat, a pyrazolone-triazole compound, exhibits high selectivity for PHD2, the isoform most critical for HIF-α stabilization [31]. Its selectivity derives from unique structural features: a bidentate interaction with the active Fe^2+^ ion, π–π stacking with Tyr303 within the 2-OG binding pocket, a rigid tricyclic core that improves shape complementarity, and absence of a glycineamide side chain that reduces off-target interactions [31]. This selectivity profile reduces off-target effects and contextualizes molidustat's design as an improvement over earlier non-selective inhibitors.

Despite their clinical success, the potential for HIF-independent effects of HIF-PHIs remains poorly characterized. The distinct molecular structures and PHD isoform selectivity profiles of roxadustat and molidustat make them ideal candidates for identifying non-canonical mechanisms. To systematically investigate these effects, we generated a ΔHIF1A/ΔEPAS1 double-knockout cell model eliminating both HIF-1α and HIF-2α. Our results demonstrate that roxadustat and molidustat amplify distinct phenotypic alterations in this HIF-null background, confirming HIF-1/2α-independent biological actions. Subsequent RNA sequencing and pathway enrichment analysis revealed extensive HIF-independent transcriptional reprogramming induced by both compounds.

These findings have two critical implications. First, they highlight mechanisms that could drive adverse drug safety outcomes unrelated to canonical HIF signaling. Second, they uncover novel biological pathways that could expand the clinical applications of these drugs beyond anemia and metabolic disorders. By mapping the HIF-independent landscapes of these pharmacologically distinct agents, this work establishes a foundation for improving their risk-benefit profiles and identifying new therapeutic opportunities.

## 2 Results

### 2.1 Structural comparison between roxadustat and molidustat: Elongated versus compact

**Fig. 1:**
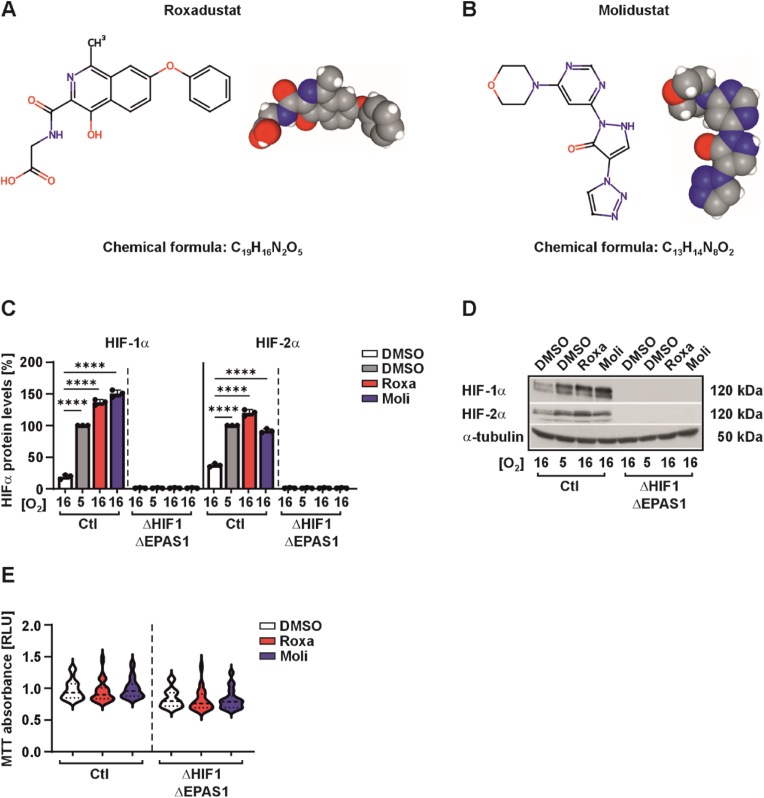
Structural comparison of HIF-PH inhibitors and ΔHIF1ΔEPAS1 cell line validation **(A**) Roxadustat (chemical formula: C_19_H_16_N_2_O_5_; IUPAC name: 2-[(4-hydroxy-1-methyl-7-phenoxyisoquinoline-3-carbonyl)amino]acetic acid). Left: 2D structural formula; right: 3D ball-and-stick model. **(B)** Molidustat (chemical formula: C_13_H_14_N_8_O_2_; IUPAC name: 2-(6-morpholin-4-ylpyrimidin-4-yl)-4-(1H-1,2,3-triazol-1-yl)-1H-pyrazol-3-one). Left: 2D structural formula; right: 3D ball-and-stick model. Red: Oxygen; Blue: Nitrogen; Grey: Carbon; White: Hydrogens. (Source: PubChem, NCBI). **(C)** PC-3 control (Ctl) and PC-3 ΔHIF1ΔEPAS1 cells were seeded on 6 cm plates and cultured for 24 h. Thereafter, cells were treated with roxadustat (Roxa; 10 μM) and molidustat (Moli; 5 μM), further cultured under normoxia (16% O_2_) and hypoxia (5% O_2_) for 16 h and then harvested. In each experiment the protein levels under 5% O_2_ were set to 100%. Data are mean±SD from 3 independent experiments. The statistical significance of differences was determined using ordinary one-way ANOVA. ∗∗∗∗p < 0.0001. **(D)** Representative Western blot analysis. 100 μg of total protein lysate was analyzed with antibodies against HIF-1α, HIF-2α and α-tubulin. **(E)** PC-3 control (Ctl) and PC-3 ΔHIF1ΔEPAS1 cells were seeded onto 24-well plates at a density 4 x 10^4^ cells/well and cultured under normoxia (16% O_2_). After 24 h cells were treated with either roxadustat (Roxa; 10 μM) or molidustat (Moli; 5 μM) for 24 h; MTT-formazane formation was measured at 570 nm using a Tecan plate reader.

To understand whether the clinically approved HIF-PHIs, roxadustat and molidustat, cause effects that cannot be explained by HIF-1α/HIF-2α stabilization alone, we assessed their structure. Both drugs have distinct molecular structures and PHD isoform selectivity profiles. Roxadustat has a more extended geometry featuring an isoquinoline core with a phenoxy substituent and mimics 2-OG binding with a glycineamide side chain that interacts with the active site of HIF-PHIs and other 2-OG-dependent oxygenases, which makes selectivity challenging [27]. Further, the glycinamide group is sensitive to metabolic degradation (Fig. 1A) [26]. Conversely, molidustat employs a more compact structure, characterized by the absence of the glycineamide side chain and the presence of a distinctive scaffold comprising triazole, pyrazolone, pyrimidine, and morpholine rings. This compact structure correlates with molidustat's higher potency and selectivity for PHD2, as evidenced by an IC50 of 7 nM versus roxadustat's (27 nM). It also presents a potentially more metabolically stable alternative (Fig. 1B) [31].

### 2.2 Roxadustat and molidustat affect HIF-1α and HIF-2α as well as their target genes

To dissect the role of roxadustat and molidustat independent of the canonical HIF-1/2α pathway, we generated HIF-1/2α double knockout (ΔHIF1ΔEPAS1) PC-3 cells by using CRISPR/Cas9-mediated non-homologous end-joining (NHEJ). Western blot analysis confirmed complete absence of HIF-1/2α proteins in ΔHIF1ΔEPAS1 cells versus scrambled controls (Ctl) under both normoxic (16% O_2_) and hypoxic (5% O_2_) conditions (Fig. 1C and D). Both hypoxia and treatment with either roxadustat or molidustat upregulated HIF-1/2α protein levels exclusively in Ctl cells but not in ΔHIF1ΔEPAS1 cells (Fig. 1C and D). To rule out toxicity, a cell viability assay determined the optimal concentrations of roxadustat and molidustat. The selected doses of 10 μM roxadustat and 5 μM molidustat did not significantly affect cell viability (Fig. 1E), whereas higher concentrations of both HIF-PH inhibitors reduced viability (Suppl. Fig. 1A and 1B). To further validate the functional knockout, we analyzed the expression of canonical HIF targets. In Ctl cells, hypoxia, roxadustat, and molidustat upregulated the protein levels of CAIX and BNIP3, two well-known downstream targets of HIFs, whereas ΔHIF1ΔEPAS1 cells showed either weak or undetectable expression of these targets under all conditions, confirming the successful generation of the double knockout (Suppl. Fig. 2A). Further, an EPO-HRE luciferase reporter construct was activated by hypoxia, roxadustat, and molidustat in HEK-293 cells (Suppl. Fig. 2B).

### 2.3 Roxadustat and molidustat affect cell proliferation and colony formation

**Fig. 2:**
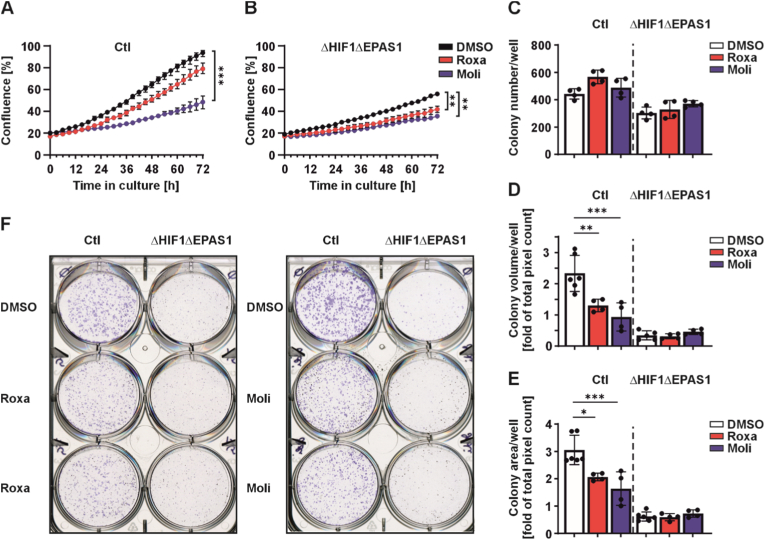
**Roxadustat and molidustat affect cell proliferation and colony formation.** Live cell proliferation analysis of control (Ctl) cells **(A)** and ΔHIF1ΔEPAS1 cells **(B)** treated with roxadustat (Roxa; 10 μM) or molidustat (Moli; 5 μM). Data are mean±SD (n = 4). Statistical significance was determined by ordinary one-way ANOVA. ∗∗p < 0.01, ∗∗∗∗p < 0.0001. **(C,D,E)** Cells were seeded onto 6-well plates at a density of 2000 cells/well, allowed to settle for 24 h, and then treated with roxadustat (Roxa; 10 μM) or molidustat (Moli; 5 μM). The cells were cultured for an additional 10 days. Every second day, medium was replaced with fresh inhibitors. Afterwards, cells were fixed with 4% paraformaldehyde and stained with crystal violet. **(C)** Colony number, **(D)** colony volume, and **(E)** colony area were analyzed. Data are mean±SD from 4 independent experiments. Statistics: Ordinary one-way ANOVA. ∗p < 0.05, ∗∗p < 0.01, ∗∗∗p < 0.001 ∗∗∗∗p < 0.0001. **(F)** Representative images of stained colonies.

To better understand the influence of roxadustat and molidustat on ΔHIF1ΔEPAS1 cells, we first performed proliferation and colony formation assays. Treatment with roxadustat significantly inhibits cell proliferation in cells lacking HIF-1/2α, while control (Ctl) cells remain largely unaffected. Although control (Ctl) cells do not exhibit a statistically significant reduction in proliferation, there is a noticeable trend toward slower proliferation upon roxadustat treatment, suggesting a mild inhibitory effect (Fig. 2A and B). In contrast, treatment with molidustat has a much more pronounced inhibitory effect on cell proliferation in control cells compared to the ΔHIF1ΔEPAS1 cells (Fig. 2A and B). The inhibition of cell proliferation is further enhanced by elevated doses of both roxadustat and molidustat. In addition, at higher concentrations, the inhibitory effect of roxadustat is more prominent in the double knockout cells, whereas molidustat exhibits a stronger inhibitory effect on the proliferation of the control cells (Suppl. Fig. S3A–S3D). This opposing pattern of sensitivity suggests that the molecular pathways affected by roxadustat and molidustat may be differentially regulated in ΔHIF1ΔEPAS1 cells versus control cells. It is noteworthy that cells deficient in HIF-1/2α appear to proliferate at a significantly slower rate compared to the control cells (Fig. 2A and B). This is consistent with previous findings demonstrating that mouse embryonic fibroblasts lacking HIF-1α also show reduced proliferation compared to their respective control cells [32].

Next, we investigated the effects of roxadustat and molidustat on the colony-forming ability of ΔHIF1ΔEPAS1 cells. Treatment with either roxadustat or molidustat did not significantly affect the number of colonies formed by both control (Ctl) and ΔHIF1ΔEPAS1 cells (Fig. 2C and F). Interestingly, when analyzing the colony volume and colony area, both inhibitors reduced the volume and the area of control cell colonies (Fig. 2D–F). However, neither roxadustat nor molidustat had any impact on the colony volume or colony area in ΔHIF1ΔEPAS1 cells (Fig. 2D–F). It is worth noting that ΔHIF1ΔEPAS1 cells initially exhibited a reduced colony-forming capacity compared to control cells, likely due to their lower proliferation rate. The increase in colony number but no change in colony volume or colony area in ΔHIF1ΔEPAS1 cells upon treatment with HIF-PH inhibitors may reflect a compensatory survival mechanism in the absence of HIF-1/2α rather than altered cell-cell interaction since no differences in cell adhesion was observed (Suppl. Fig. S4). These survival mechanisms may involve PI3K/AKT/mTOR and NF-κB signaling for anti-apoptotic responses and metabolic homeostasis [33,34], MYC-driven rearrangement of glycolysis and glutamine metabolism [35], and AMPK-mediated autophagy to maintain energy balance [36,37], underscoring the adaptability of cells in the absence of the canonical hypoxia signaling pathway.

Although these results indicate that the anti-proliferative effects of roxadustat and molidustat are independent of HIF-1α/2α, we next asked whether PHD inhibition is required or whether PHD-independent mechanisms contribute. To address this, we used a PHD1/2/3 triple knockout cell line (ΔPHD1,2,3), enabling us to determine if the drugs require PHD1–3 to cause the observed anti-proliferative phenotypes.

Both roxadustat and molidustat significantly reduced BrdU incorporation in both control and ΔPHD1,2,3 cells, although ΔPHD1,2,3 cells exhibited higher basal proliferation (Suppl. Fig. S5). Similarly, both drugs reduced colony number and size in both genotypes, with molidustat showing greater potency. Notably, ΔPHD1,2,3 cells formed larger colonies under basal conditions, suggesting a proliferative advantage in the absence of PHD activity (Suppl. Fig. S5).

These findings demonstrate that the anti-proliferative effects of roxadustat and molidustat persist even in the absence of functional PHD1-3, indicating PHD-independent mechanisms. Together, the consistent phenotypes in both ΔHIF1ΔEPAS1 and ΔPHD1,2,3 models strongly suggest that roxadustat and molidustat exert biological effects beyond the canonical PHD–HIF axis. This implies that additional targets such as other 2-OGDDs or hydroxylation-sensitive regulatory pathways may contribute to their observed effects.

### 2.4 Roxadustat and molidustat affect cell cycle and apoptosis

**Fig. 3:**
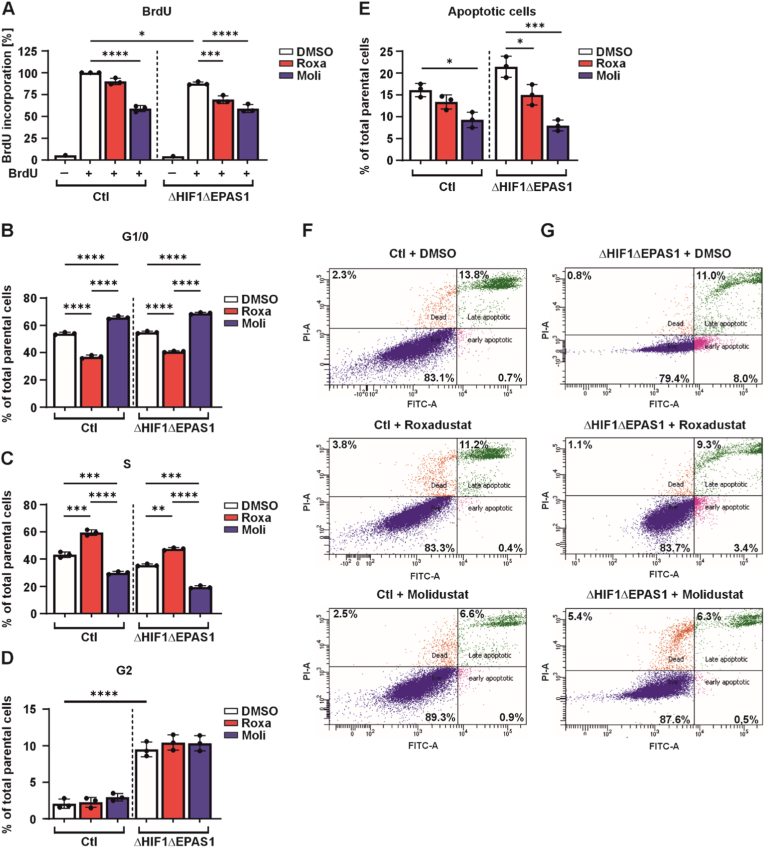
Roxadustat and molidustat affect the cell cycle and apoptosis PC-3 control (Ctl) and ΔHIF1ΔEPAS1 cells were treated with roxadustat (Roxa; 10 μM) or molidustat (Moli; 5 μM) for 24 h and then harvested for further analysis. **(A)** BrdU incorporation. **(B,C,D)** Cell cycle distribution: G1/0 phase **(B)**, S phase **(C)**, and G2 phase **(D)**. **(E)** Quantification of total apoptosis. All data are mean±SD from 3 independent experiments. Statistics: Ordinary one-way ANOVA. ∗p < 0.05, ∗∗p < 0.01, ∗∗∗p < 0.001 ∗∗∗∗p < 0.0001. **(F,G)** Representative histograms of apoptosis, as assessed by Annexin-V/PI staining and measured by flow cytometry.

The above findings suggest that roxadustat and molidustat may play a role in both cell proliferation and colony-forming ability. To validate our live-cell proliferation results, first we assessed DNA synthesis by measuring bromodeoxyuridine (BrdU) incorporation. Consistent with the live-cell proliferation measurement, treatment with roxadustat and molidustat significantly reduced BrdU incorporation in both control (Ctl) and ΔHIF1ΔEPAS1 cells (Fig. 3A). Notably, ΔHIF1ΔEPAS1 cells exhibited markedly lower BrdU incorporation compared to control cells, further confirming their reduced proliferative capacity (Fig. 3A). Next, we tested whether roxadustat or molidustat affect the cell cycle and found that treatment with both roxadustat and molidustat revealed differential effects during the S and G1/0 phase of the cell cycle, indicating phase-specific modulation of cellular processes (Fig. 3B and C). Notably, these differential effects were consistent in both control (Ctl) and ΔHIF1ΔEPAS1 cells, suggesting that the influence of both HIF-PH inhibitors on the cell cycle progression is independent of HIF-1/2α (Fig. 3B and C). In contrast, neither roxadustat nor molidustat had a significant impact on the G2 phase. However, the G2 phase accumulation was increased in ΔHIF1ΔEPAS1 cells compared to the control (Ctl) cells, suggesting a HIF-1/2α-dependent alteration in cell cycle dynamics by modulating the G2 phase (Fig. 3D).

Following flow cytometry in control (Ctl) cells revealed that treatment with molidustat for 24 h decreased the number of apoptotic cells, whereas roxadustat had no significant influence on apoptosis, although there is a tendency showing that roxadustat also reduced the number of apoptotic cells in control (Ctl) cells (Fig. 3E and F). Similarly, when treating ΔHIF1ΔEPAS1 cells with roxadustat or molidustat, they follow the same pattern as the control cells (Fig. 3E and G). Thus, the obtained data suggest that the anti-apoptotic effects of HIF-PH inhibitors are independent of HIF-1/2α.

### 2.5 Roxadustat and molidustat cause changes in mitochondrial function, oxidative stress, lysosomal activity, and lipid accumulation

**Fig. 4:**
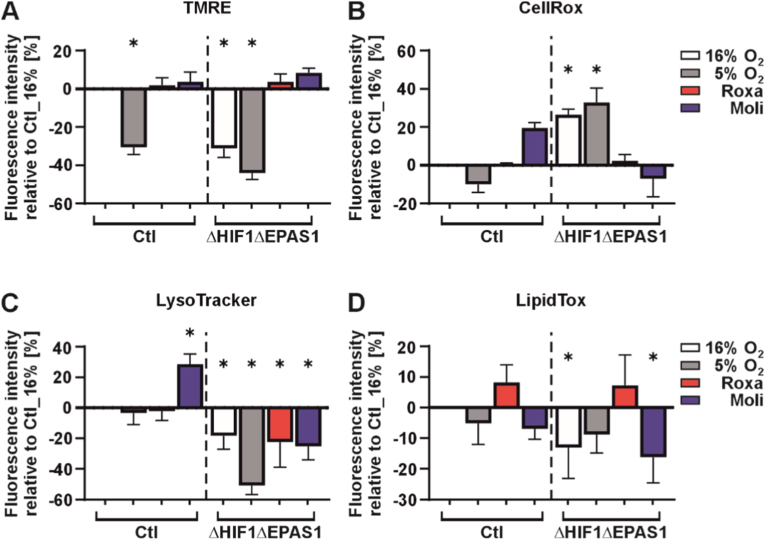
Roxadustat and molidustat modulate cell metabolism and organelle function independently of HIF-1α and HIF-2α. PC-3 control (Ctl) and ΔHIF1ΔEPAS1 cells were grown on 96-well plates and treated with either roxadustat (Roxa; 10 μM) or molidustat (Moli; 5 μM) and cultured under normoxia and hypoxia for 24 h. **(A)** TMRE analysis, **(B)** CellROX® measurement, **(C)** LysoTracker™ Red measurement, and **(D)** LipidTOX™ measurement. All measurements were analyzed and quantified with the Operetta high-content imaging system. In each experiment, the fluorescence intensity of control cells (Ctl) cultured under 16% O_2_ were set to 0. Statistical differences were calculated by using Student *t*-test, with error probabilities of p < 0.05 considered to be significant.

To get deeper insights into the metabolic, organelle, and redox adaptations driven by HIF-PH inhibitors but independent of HIF-1/2α, we analyzed mitochondrial function, autophagy, oxidative stress response, and lipid metabolism in high-throughput screening assays. Since it is well established that HIFs, in particular HIF-1, play a significant role in regulating mitochondrial function, we first assessed the mitochondrial membrane potential. Under hypoxic conditions, control (Ctl) cells exhibited a ∼30% reduction in mitochondrial membrane potential. However, treatment with roxadustat or molidustat completely abolished this reduction (Fig. 4A). Interestingly, ΔHIF1ΔEPAS1 cells cultured under hypoxia did not show a significant decrease in mitochondrial membrane potential compared to those cells maintained under normoxia and that the baseline of the mitochondrial membrane potential in ΔHIF1ΔEPAS1 cells is about ∼30% lower than that observed in control (Ctl) cells. Nevertheless, treatment with roxadustat or molidustat also increased the mitochondrial membrane potential by approximately 30% in ΔHIF1ΔEPAS1 cells, relative to their normoxic counterparts (Fig. 4A). These findings suggest that roxadustat and molidustat can modulate mitochondrial membrane potential independently of HIF-1/2α.

Given the critical role of mitochondria in maintaining reactive oxygen species (ROS) homeostasis, we next used the Operetta high-content platform and the CellROX fluorogenic assay to assessed ROS production in both control (Ctl) and ΔHIF1ΔEPAS1 cells. In control cells, hypoxia had no significant effect on ROS levels compared to normoxic conditions. Similarly, treatment with roxadustat or molidustat did not alter ROS levels (Fig. 4B). In contrast, ROS levels were elevated in ΔHIF1ΔEPAS1 cells under both normoxia and hypoxia compared to control cells (Fig. 4B). Importantly, treatment with either roxadustat or molidustat resulted again in a reduction of ROS levels relative to normoxic control cells (Fig. 4B). These results could be confirmed in an orthogonal assay by measuring ROS levels with DCF-DA (Suppl. Fig. S6). Thus, these data indicate that both HIF-PH inhibitors may have a potential HIF-1/2α-independent antioxidant effect.

Since lysosomes, like mitochondria, play a vital role in maintaining cellular homeostasis, we investigated whether roxadustat or molidustat affects the lysosomal compartment. To assess this, we stained both control (Ctl) and ΔHIF1ΔEPAS1 cells with LysoTracker Red. In control cells, only molidustat treatment led to a detectable increase in fluorescence intensity by about ∼25%, while neither hypoxia nor roxadustat had any effect (Fig. 4C). In contrast, ΔHIF1ΔEPAS1 cells cultured under hypoxia showed a significant reduction in fluorescence intensity, which was completely rescued by treatment with either roxadustat or molidustat (Fig. 4C). Hence, these results suggest that both HIF-PH inhibitors can restore lysosomal functions through mechanisms that are independent of HIF-1/2α.

Given the evidence that roxadustat and molidustat can modulate lysosomal functions independently of HIF-1/2α and that lysosomes are closely linked to lipid metabolism, we investigated whether lipid processes can also be influenced. We found that neither hypoxia nor roxadustat nor molidustat exerted any influence on the lipid accumulation in the control (Ctl) cells (Fig. 4D). In ΔHIF1ΔEPAS1 cells an increase in lipid accumulation was only observed upon roxadustat treatment compared to the normoxic control (Fig. 4D). Thus, these data indicate that roxadustat may have a possible HIF-1/2α-independent role in lipid metabolism under specific cellular conditions. Collectively, roxadustat and molidustat may influence cellular processes, including mitochondrial function, oxidative stress response, lysosomal activity, and lipid metabolism independently of HIF-1/2α. Furthermore, roxadustat shows distinct effects in ΔHIF1ΔEPAS1 cells, including reduced ROS levels and increased lipid accumulation, while molidustat notably enhances mitochondrial membrane potential and lysosomal function in control cells. These findings highlight compound-specific, HIF-1/2α-independent roles in cellular homeostasis.

### 2.6 HIF-PH inhibitors modulate wound healing, angiogenesis, and tumor formation

**Fig. 5:**
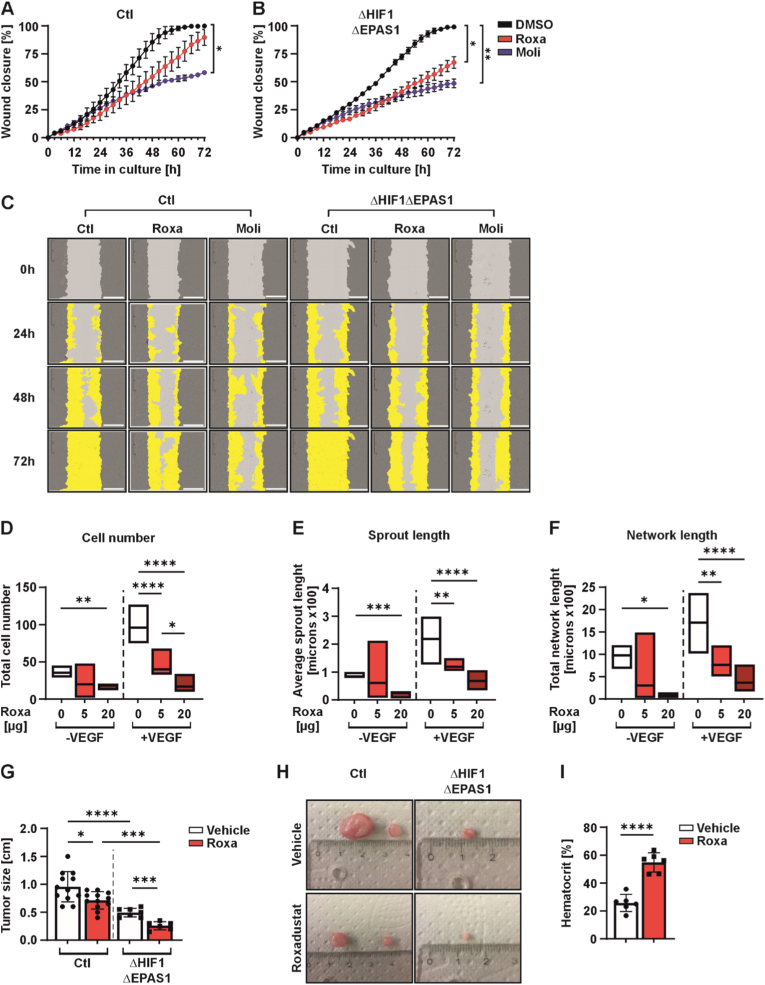
HIF-PH inhibitors affect migration, angiogenesis and tumor formation. (**A, B**) Live cell wound closure analysis of PC-3 control (Ctl) and ΔHIF1ΔEPAS1 cells treated with roxadustat (Roxa; 10 μM) and molidustat (Moli; 5 μM). Data are mean±SD from 3 independent experiments. **(C)** Representative wound closure images of PC-3 control (Ctl) and ΔHIF1ΔEPAS1 cells. **(D,E,F)** Quantification of the angiogenesis assay in HUVEC cells upon roxadustat (Roxa) treatment for 7 days: Cell number **(D)**, sprout length **(E)**, and network length **(F)**; **(G)** Quantification of the tumor size in PC-3 control (Ctl) and ΔHIF1ΔEPAS1 cells upon roxadustat (Roxa) treatment in nude mice (6 animals per group). **(H)** Representative images of tumors formed by PC-3 control (Ctl) and ΔHIF1ΔEPAS1 cells in nude mice. **(I)** Hematocrit quantification in nude mice upon roxadustat (Roxa) treatment (6 mice per group). In all experiments, statistical significance was determined using an ordinary one-way ANOVA. ∗p < 0.05, ∗∗p < 0.01, ∗∗∗p < 0.001, ∗∗∗∗p < 0.0001.

To provide a more comprehensive understanding of the observed alterations in proliferation and metabolic processes driven by roxadustat and molidustat independently of HIF-1/2α, we conducted additional experiments. First, we performed wound healing assay in control (Ctl) and ΔHIF1ΔEPAS1 cells and found that molidustat significantly diminished the wound healing ability of both control (Ctl) and ΔHIF1ΔEPAS1 cells by about ∼50%. In contrast, treatment with roxadustat led to a substantial reduction in wound closure, with a decrease of approximately 40% observed only in ΔHIF1ΔEPAS1 cells (Fig. 5A–C). Of note, roxadustat exhibited a clear visible inhibitory effect on the wound closure capacity of control (Ctl) cells, though no substantial difference was observed after 72 h (Fig. 5A and C).

To investigate the effects of HIF-PH inhibitors in a non-cancerous cell line, we carried out an angiogenesis assay using human umbilical vein endothelial cells (HUVECs). Given the critical role of angiogenesis in both physiological and pathological processes, investigating these effects would lead to a broader understanding of HIF-PH inhibitors for therapeutic implications. Here, we focused specifically on roxadustat, as it has already approved for clinical use and its vascular effects are particularly relevant for patients undergoing long-term treatment. We found that treatment of HUVECs with 20 μM roxadustat significantly reduced cell number, sprout length, and network length under baseline conditions in the absence of VEGF stimulation. In contrast, 5 μM roxadustat had no substantial effect on these angiogenic parameters after 7 days in culture (Fig. 5D–F). Under pro-angiogenic conditions induced by VEGF, both 5 μM and 20 μM roxadustat significantly reduced cell number, sprout length, and network length (Fig. 5D–F). Interestingly, after 3 days in culture, roxadustat at both concentrations affected cell number under baseline conditions, while sprout and network lengths remained unchanged compared to untreated controls (Suppl. Fig. S7). Upon VEGF stimulation, both concentrations reduced cell number and network length, but only 5 μM roxadustat significantly decreased sprout length (Suppl. Fig. S7). Notably, these findings suggest that low-dose roxadustat (5 μM) may be more effective at inhibiting angiogenic factors during short-term exposure, whereas higher doses (20 μM) exert stronger effects over longer periods in HUVECs.

The above in vitro data suggest that roxadustat exerts its anti-angiogenic and anti-cell migration effects in a dose- and time-dependent manner, even in the absence of HIF-1/2α, indicating a role of roxadustat independent of the canonical HIF pathway. To further explore the physiological and pathological relevance of these effects, we next evaluated the impact of HIF-PH inhibitors *in vivo* using a xenograft model in nude mice. Therefore, we injected subcutaneously PC-3 control (Ctl) versus ΔHIF1ΔEPAS1 cells into the flanks of 12 female athymic immune-deficient nude mice. In each mouse, two injections of the same cell type were administered per flank (in total 4 injections per mouse). After 5 days, mice were randomly divided into two groups and received three weekly intraperitoneal injections over a 4-week period: one group was treated with roxadustat (20 mg/kg body weight), while the control group received the corresponding solvent without roxadustat. Even though both cell lines were able to form tumors, it was observed that only half of the injections of the ΔHIF1ΔEPAS1 cells resulted in the formation of a tumor and it was obvious that the tumors formed from these cells were of smaller size compared to the arisen tumors from control (Ctl) cells (Fig. 5G and H). The administration of roxadustat to the mice resulted in a substantial decline in the size of tumors from both cell lines when compared to the vehicle control group (Fig. 5G and H). To confirm the efficacy of roxadustat treatment in the mice, hematocrit levels were measured on the day of necropsy. The average hematocrit in vehicle-treated mice was approximately 25.7%, while roxadustat-treated mice exhibited a significantly elevated hematocrit average of 54.9%, indicating successful treatment (Fig. 5I). Thus, these findings suggest that roxadustat may have the potential to inhibit cell proliferation as well as tumor growth independently of HIF-1/2α.

### 2.7 Transcriptomic analysis reveals differential gene expression profiles upon roxadustat treatment independently of HIF-1α and HIF-2α

**Fig. 6:**
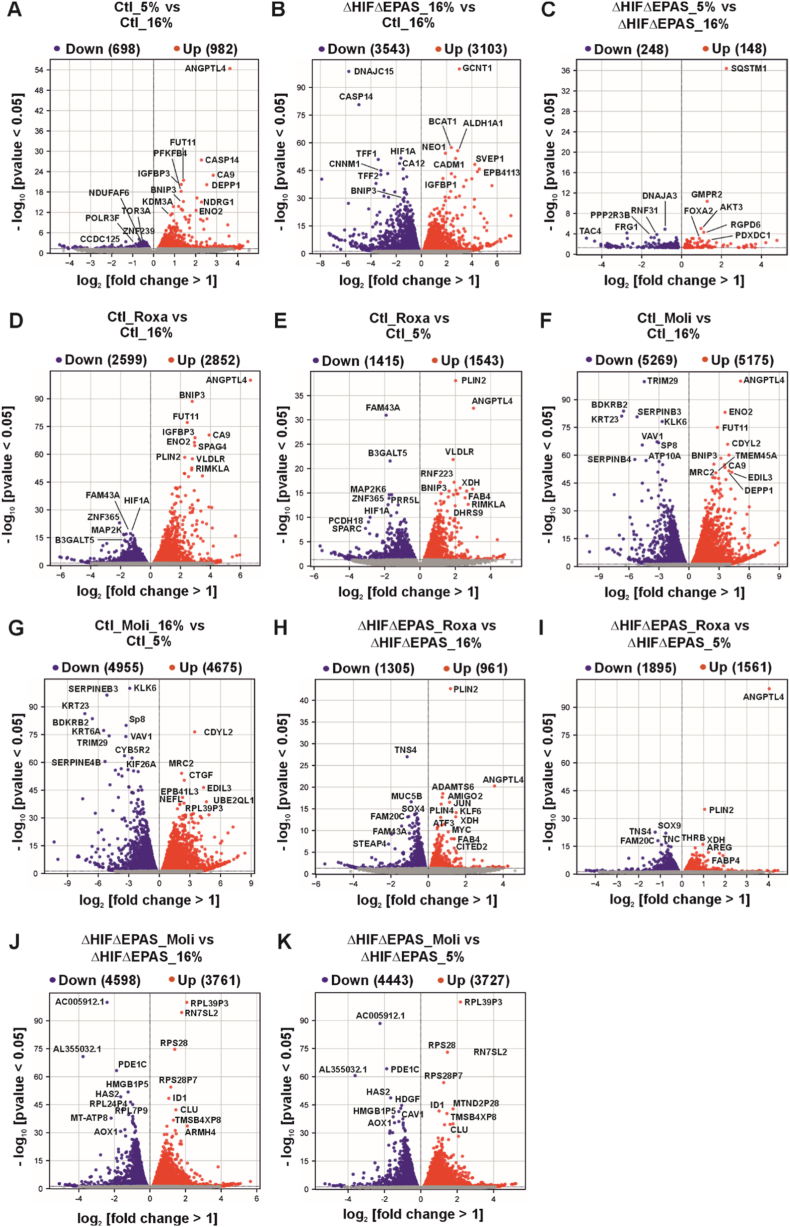
Volcano plots for differential gene expression analysis of RNAseq results. Each dot in all graphs represents a differentially expressed gene. Red dots represent genes with increased expression and blue dots represent genes with decreased expression. **(A)** Control (Ctl) 5%O_2_ versus 16%O_2_. **(B)** ΔHIF1ΔEPAS1 16%O_2_ versus control (Ctl) 16%O_2_. **(C)** ΔHIF1ΔEPAS1 5%O_2_ versus ΔHIF1ΔEPAS1 16%O_2_. **(D)** Control (Ctl) roxadustat (Roxa) versus 16%O_2_. **(E)** Control (Ctl) roxadustat (Roxa) versus 5%O_2_. **(F)** Control (Ctl) molidustat (Moli) versus 16%O_2_. **(G)** Control (Ctl) molidustat (Moli) versus 5%O_2_. **(H)** ΔHIF1ΔEPAS1 roxadustat (Roxa) versus ΔHIF1ΔEPAS1 16%O_2_. **(I)** ΔHIF1ΔEPAS1 roxadustat (Roxa) versus ΔHIF1ΔEPAS1 5%O_2_. **(J)** ΔHIF1ΔEPAS1 molidustat (Moli) versus ΔHIF1ΔEPAS1 16%O_2_. **(K)** ΔHIF1ΔEPAS1 molidustat (Moli) versus ΔHIF1ΔEPAS1 5%O_2_.

To gain deeper insight into the molecular mechanisms underlying the pronounced functional differences observed between PC-3 control and ΔHIF1ΔEPAS1 cells through various assays, we performed an unbiased high-throughput RNA sequencing (RNAseq) to possibly identify signaling pathways, gene regulatory networks, or cellular processes that are regulated by roxadustat and molidustat independently of the canonical HIF pathway. To this end, PC-3 control (Ctl) and ΔHIF1ΔEPAS1 cells were treated with both roxadustat or molidustat and further cultured for 16 h either under normoxia (16% O_2_) or hypoxia (5% O_2_). Thereafter, profiling for global transcriptomic analysis using RNAseq was performed. Correlation analysis of RNA-seq differential expression patterns showed that molidustat-treated samples had a weak similarity to other treatment groups in both control (Ctl) and ΔHIF1ΔEPAS1 cells. In contrast, samples treated with normoxia, hypoxia, and roxadustat clustered closely together (Suppl. Fig. S8A and S8B). To systematically dissect the transcriptional effects of roxadustat and molidustat treatment independently of HIF-1/2α, we performed a multi-step filtering strategy. First, we identified hypoxia-regulated genes by comparing control (Ctl) cells under normoxia with those under hypoxia (Fig. 6A). To find genes that are likely regulated independently of HIF-1/2α, we compared control (Ctl) cells versus ΔHIF1ΔEPAS1 cells cultured under normoxic conditions (Fig. 6B). This gene list includes both hypoxia-responsive genes and genes not responsive to hypoxia but still dependent on HIF-1/2α, possibly due to their basal activity. To verify the ΔHIF1ΔEPAS1 effect on hypoxia, we compared the double knockout cells cultured under normoxia to those under hypoxia and found minimal differential gene expression for hypoxia-responsive genes in ΔHIF1ΔEPAS1 cells (Fig. 6C) unlike the comparison control (Ctl) cells cultured under normoxia versus hypoxia (Fig. 6A). Next, we identified genes regulated by either roxadustat or molidustat in both control (Ctl) and ΔHIF1ΔEPAS1 cells under normoxic as well as hypoxic conditions. Therefore, we compared control (Ctl) cells cultured under normoxia and hypoxia with those treated with either roxadustat (Fig. 6D and E) or molidustat (Fig. 6F and G). The same comparison was performed with ΔHIF1ΔEPAS1 cells treated with either roxadustat (Fig. 6H and I) or molidustat (Fig. 6J and K).

**Fig. 7:**
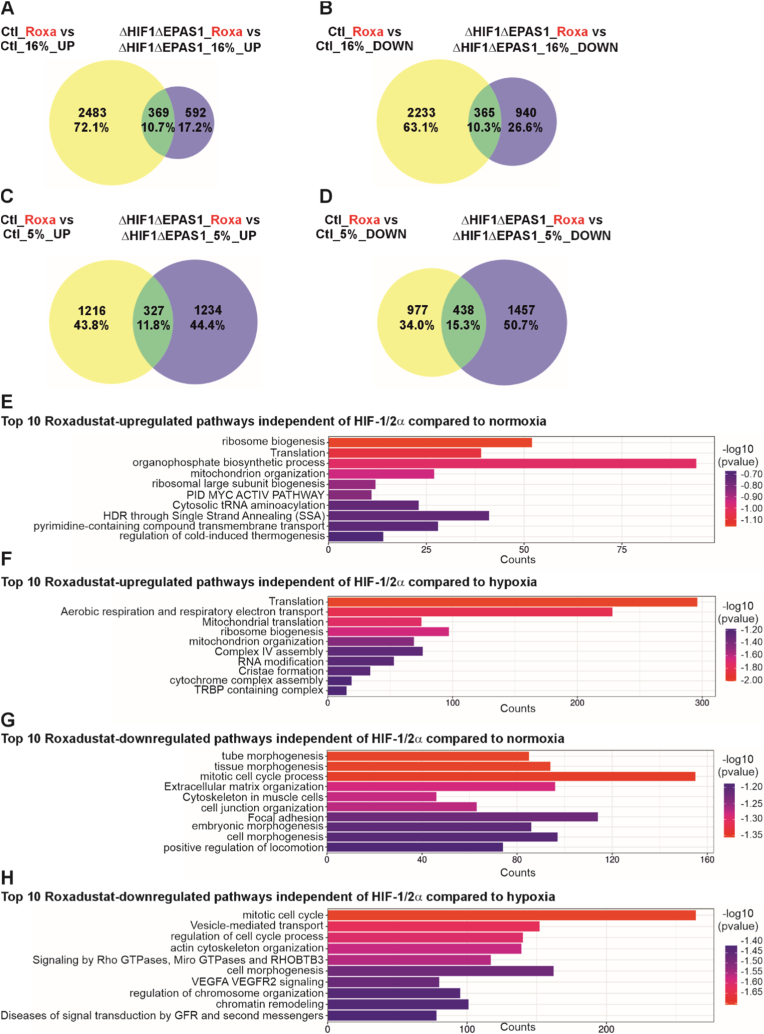
Proportional Venn diagrams and Gene Ontology (GO) pathway analysis of ΔHIF1ΔEPAS1 cells compared to control cells upon roxadustat treatment. **(A)** Upregulated genes in the control group (Ctrl) with roxadustat (Roxa) at 16% O_2_ versus the ΔHIF1ΔEPAS1 group with roxadustat (Roxa) at 16% O_2_. **(B)** Downregulated genes: control (Ctl) vs. roxadustat (Roxa) at 16% O_2_ in ΔHIF1ΔEPAS1 cells. **(C)** Upregulated genes in the control group (Ctl) with roxadustat (Roxa) at 5% O_2_ versus the ΔHIF1ΔEPAS1 group with roxadustat (Roxa) at 5% O_2_. **(D)** Down-regulated genes: control (Ctrl) roxadustat (Roxa) at 16% O_2_ versus ΔHIF1ΔEPAS1 roxadustat (Roxa) at 16% O_2_. **(E)** Roxadustat-upregulated pathways in ΔHIF1ΔEPAS1 cells versus 16% O_2_ or 5% O_2_. **(F)** Down-regulated pathways by roxadustat in ΔHIF1ΔEPAS1 cells vs. 16% O_2_ or 5% O_2_.

Next, we conducted a comparative analysis of gene expression changes in response to treatment with roxadustat in both control (Ctl) cells and ΔHIF1ΔEPAS1 cells. Specifically, we quantified the number and percentage of genes that were upregulated or downregulated following treatment, relative to cells cultured under normoxic conditions. Upon roxadustat treatment, 10.7% of upregulated genes and 10.3% of downregulated genes were shared between control (Ctl) and ΔHIF1ΔEPAS1 cells (Fig. 7A and B). The majority of roxadustat-responsive genes remained regulated in control cells, with 72.1% upregulated and 63.1% downregulated genes, whereas in ΔHIF1ΔEPAS1 cells, only 17.2% upregulated and 26.6% genes were downregulated (Fig. 7A and B). This divergence became more pronounced when comparing roxadustat-treated cells to those cultured under hypoxic conditions. Although the percentage of overlapping genes remained unchanged (Fig. 7C and D), the proportion of uniquely regulated genes in ΔHIF1ΔEPAS1 cells increased to 44.4% upregulated and 50.7% downregulated genes, while in control cells, these values decreased to 43.8% and 34.0%, respectively (Fig. 7C and D). A comparison of all differentially expressed genes under various conditions is shown in Suppl. Fig. S9A and S9B. These findings suggest that the knockout of HIF-1α and HIF-2α significantly alters the gene regulatory effects of roxadustat, particularly under hypoxia, potentially uncovering alternative or compensatory mechanisms of gene regulation in ΔHIF1ΔEPAS1 cells.

To ensure that the regulatory effects of roxadustat treatment is independent of HIF-1/2α, we excluded all genes influenced by them. Therefore, we combined hypoxia-responsive genes and genes altered by HIF-1/2α under normoxic conditions, since these two sets together represent all genes affected by HIF-1/2α. To isolate the roxadustat-specific regulation, these HIF-1/2α-regulated genes are then excluded from the intersection-regulated genes (Fig. 7A–D). This filtering ensured that the observed effects of roxadustat are not confounded by the presence or absence of HIF-1/2α. The 20 most significant up- and down-regulated genes upon roxadustat treatment independent of HIF-1/2α are shown in Suppl. Fig. S10A and S10B and Table S1. To identify the signaling pathways modulated by differentially expressed genes independent of HIF-1/2α upon roxadustat treatment, a Gene Ontology (GO) enrichment analysis was performed using Metascape (http://metascape.org). Among the signaling pathways most strongly upregulated by roxadustat, we identified a significant overlap when compared to normoxic or hypoxic conditions, including ribosome biogenesis, translation regulation, and mitochondrial organization. In addition, specific signaling pathways were found to be selectively upregulated in comparison of either normoxia or hypoxia (Fig. 7E and F, Suppl. Data file 1: DEGs_GO_Roxa). It is noteworthy when compared to both oxygen conditions, the predominant roxadustat-induced pathways independently of HIF-1/2α were associated with RNA modification, ribosome function, and mitochondrial processes. Thus, these findings suggest a consistent transcriptional response targeting cellular biosynthesis and metabolic mechanisms.

Compared to normoxic conditions in control cells, the signaling pathways most strongly downregulated by roxadustat in ΔHIF1ΔEPAS1 cells are cellular processes primarily focused on structural development and tissue integrity. This involves the organization of the extracellular matrix, cell junctions, and morphogenetic pathways. Conversely, hypoxic conditions elicit adaptive responses that promote survival and stress management, encompassing chromatin remodeling, vesicle-mediated transport, and angiogenic signaling via VEGFA–VEGFR2 (Fig. 7G and H, Suppl. Data file 1: DEGs_GO_Roxa). These findings underscore that roxadustat profoundly alters cellular functions, both in signaling pathways associated with cell growth and structural organization, and in those involved in signal transduction, regulation, and stress adaptation, even in the absence of HIF-1/2α.

### 2.8 Transcriptomic analysis reveals differential gene expression profiles upon molidustat treatment independently of HIF-1α and HIF-2α

**Fig. 8:**
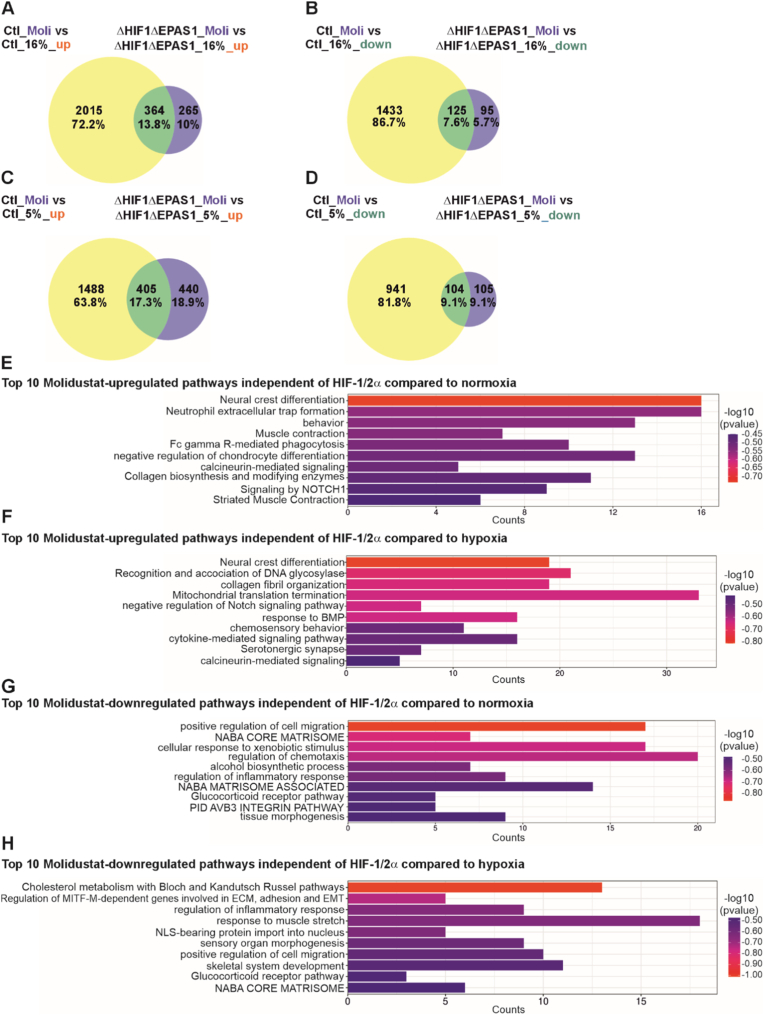
Proportional Venn diagrams and Gene Ontology (GO) pathway analysis of ΔHIF1ΔEPAS1 cells compared to control cells upon molidustat treatment. **(A)** Upregulated genes: control (Ctl), molidustat (Moli), 16% O_2_ vs. ΔHIF1ΔEPAS1, molidustat (Moli), 16% O_2_. **(B)** Down-regulated genes: control (Ctl) molidustat (Moli) at 16% O_2_ versus ΔHIF1ΔEPAS1 molidustat (Moli) at 16% O_2_. **(C)** Upregulated genes: control (Ctl), molidustat (Moli) at 5% O_2_ versus ΔHIF1ΔEPAS1 molidustat (Moli) at 5% O_2_. **(D)** Down-regulated genes: control (Ctl) molidustat (Moli) at 16% O_2_ versus ΔHIF1ΔEPAS1 molidustat (Moli) at 16% O_2_. **(E)** Molidustat-upregulated pathways in ΔHIF1ΔEPAS1 cells versus 16% O_2_ or 5% O_2_. **(F)** Molidustat-downregulated pathways in ΔHIF1ΔEPAS1 cells versus 16% O_2_ or 5% O_2_.

When treated the cells with molidustat, a small fraction of genes, 13.8% of those upregulated and 7.6% of those downregulated, were commonly regulated in both control (Ctl) and ΔHIF1ΔEPAS1 cells cultured under normoxia. Most of the gene expression changes in response to molidustat occurred in control (Ctl) cells, where 72.2% of upregulated and 86.7% of downregulated genes were observed (Fig. 8A and B). In contrast, ΔHIF1ΔEPAS1 cells showed a weaker response to molidustat treatment, with only 10.0% of genes upregulated and 5.7% downregulated compared to roxadustat treatment (Fig. 8A and B). Under hypoxic conditions, again the overall pattern shifted and although the overlap between control (Ctl) and ΔHIF1ΔEPAS1 cells remained almost similar, the number of differentially expressed genes in ΔHIF1ΔEPAS1 cells doubled, increasing to 18.9% upregulated and 9.1% downregulated genes, while the corresponding values in control (Ctl) cells dropped to 63.3% and 81.8%, respectively (Fig. 8C and D). A comparison of all differentially expressed genes under certain conditions is shown in Suppl. Fig. S9C and S9D. These results highlight that the absence of HIF-1α and HIF-2α significantly modulates the transcriptional response to molidustat, especially when compared to hypoxia, but that the majority of gene regulation still occurs in control (Ctl) cells cultured under both normoxic and hypoxic conditions. However, the increase in gene regulation observed in comparison to hypoxia in ΔHIF1ΔEPAS1 cells treated with molidustat suggests to the existence of alternative or compensatory pathways.

To isolate molidustat-specific effects, HIF-1/2α-regulated genes were excluded from the intersection-regulated genes (Fig. 8A–D). The 20 most significant up- and down-regulated genes upon molidustat treatment independent of HIF-1/2α are shown in Suppl. Fig. S11A and S11B, and Table S2. The GO enrichment analysis of the identified genes upregulated by molidustat compared to control (Ctl) cells cultured under normoxia revealed that the cells maintain structural and immune functions like muscle contraction, collagen biosynthesis, and phagocytosis, thereby reflecting active tissue maintenance. In contrast, when compared to control (Ctl) cell cultured under hypoxia, molidustat activates stress responses such as DNA repair, mitochondrial translation termination, and cytokine signaling, along with developmental shifts like NOTCH pathway suppression (Fig. 8E and F, Suppl. Data file 2: DEGs_GO_Moli). Notably, molidustat supports a wide range of homeostatic and developmental functions independent of HIF-1/2α when compared to Ctl cells cultured under normoxia, while in comparison to hypoxic conditions it triggers adaptive mechanisms that prioritize survival, differentiation, and structural remodeling.

The most downregulated pathways by molidustat when compared to control (Ctl) cells cultured under normoxia are related to migration, inflammation, and extracellular matrix (ECM) organization, including glucocorticoid signaling, xenobiotic response, chemotaxis, and integrin activity (Fig. 8G, Suppl. Data file 2: DEGs_GO_Moli). Conversely, hypoxia leads to reduced activity in cholesterol metabolism, MITF-*M*-dependent ECM and EMT regulation, nuclear import, and developmental processes like skeletal and sensory organ morphogenesis (Fig. 8H, Suppl. Data file 2: DEGs_GO_Moli). Both conditions share downregulation of key pathways such as cell migration, inflammatory response, and core matrisome signaling. Together, molidustat compromises cellular functionality by restricting mobility, dampening immune responses, and reducing the capacity to remodel tissue independently of HIF-1/2α.

## 3 Discussion

This study is the first systematic investigation of HIF–PHI effects beyond canonical HIF-1α and HIF-2α stabilization. By using a HIF1A/EPAS1 double-knockout cell model, we show that the clinically approved HIF-PHIs roxadustat and molidustat modulate cell proliferation, cell cycle progression, apoptosis, mitochondrial function, ROS homeostasis, lipid metabolism, angiogenesis, and tumor growth through HIF-independent pathways. Critically, multiple phenotypes persist in ΔHIF1ΔEPAS1 cells, indicating that PHD inhibition can engage alternative molecular routes not attributable to HIF transcriptional signaling. In addition, roxadustat and molidustat showed even anti-proliferative effects in the absence of functional PHD1-3 suggesting PHD-independent off-target mechanisms that warrant future mechanistic investigations. Together, these findings refine mechanistic understanding of HIF–PHI selectivity, address urgent safety concerns in chronic kidney disease [38,39], and provide a framework for evaluating repurposing opportunities for these drugs in oncology and metabolic disorders.

A key goal of the present work was to connect transcriptomic outputs to functional phenotypes. Across conditions, the strongest RNA-seq signals align with the cellular responses observed: (i) cell-cycle/proliferation programs (including altered expression of proliferation and cell-cycle associated genes in HIF-deficient contexts), (ii) metabolic rewiring encompassing glycolysis/pH regulation and lipid handling (e.g., CA9, PFKFB4, ALDOC/ENO2, PLIN2 and related stress programs such as BNIP3/BNIP3L and DEPP1), (iii) migration/ECM/angiogenesis-associated programs (including robust induction of ANGPTL4 and, in selected contrasts, genes such as CTGF/CCN2, EDIL3, PDPN, KDR and MRC2), and (iv) apoptosis/autophagy-related signatures (including BNIP3/BNIP3L and stress/autophagy regulators such as SESN3, SQSTM1 and ULK1 in specific settings). These phenotype-linked signatures support a focused interpretation of the RNA-seq and provide biologically grounded context for the observed drug responses in both control and ΔHIF1ΔEPAS1 cells.

### 3.1 Structural determinants of off-target activity

The distinct molecular architectures of roxadustat and molidustat provide mechanistic insight into their divergent off-target profiles. Roxadustat as a 2-OG mimetic with a glycineamide side chain and isoquinoline core, has an extended geometry that allows it to compete directly with 2-OG at the PHDs' active site. This enables pan-inhibition of PHD1–3 (IC_50_ ~ 1.3–1.4 μM). Since 2-OG is an essential co-substrate for the ∼70-member family of 2-OG-dependent dioxygenases (2-OGDDs), structural mimicry also confers non-selective binding to other 2-OGDDs, including collagen prolyl-4-hydroxylases (CP4Hs), Jumonji C domain-containing histone demethylases, TET family DNA demethylases, and γ-butyrobetaine hydroxylase (BBOX) the final enzyme in l-carnitine biosynthesis [31,[40], [41], [42]]. Consequently, careful monitoring for carnitine-dependent metabolic perturbations or epigenetic disruptions is necessary when utilizing this compound, and other 2-OG mimicking HIF-PHIs such as enarodustat or vadadustat in a clinical setting [27].

In contrast, molidustat's compact pyrazolone-triazole-pyrimidine-morpholine scaffold employs a non-2-OG-mimetic approach, relying on bidentate iron chelation via pyrazolol nitrogen atoms and specific interactions (e.g., π-stacking with Trp389 and electrostatics with Arg383). This architecture is associated with greater selectivity over non-PHD 2-OGDDs, which may reduce the risk of unintended effects on non-PHD 2-OGDDs like FIH (factor inhibiting HIF), TET enzymes, or JMJD histone demethylases [27,31,43,44].

Our observations in ΔHIF1ΔEPAS1 cells indicate that roxadustat promotes lipid accumulation, mitochondrial membrane potential changes, and biosynthetic pathway activation. These effects-align with its broad-spectrum inhibition of 2-OGDD enzymes, potentially through HIF-independent mechanisms. In parallel, molidustat's effects on differentiation and immune response pathways, despite its relative PHD selectivity, demonstrate its potential to yield pleiotropic effects beyond HIF stabilization. These outcomes may result from residual off-target 2-OGDD engagement or modulation of PHD-regulated, non-HIF substrates and signaling cascades.

### 3.2 Cell proliferation, apoptosis, and oncogenic implications

Our data indicate that roxadustat and molidustat both inhibit cell proliferation, but with differential sensitivity depending on HIF status: roxadustat showed stronger antiproliferative effects in ΔHIF1ΔEPAS1 cells, while molidustat was more effective in control cells. These findings are consistent with earlier work showing that HIF-1α depletion alters cellular responsiveness to chemotherapeutic drugs, suggesting that basal HIF-1α activity can shape drug-response phenotypes even under well-oxygenated conditions. Mechanistically, this normoxic HIF-1α function has been linked to cell-cycle control: HIF-1α knockdown increases cells in G1 and elevates p21 and p27 at both mRNA and protein levels, with p21 induction occurring at least in part via p53-independent but SP1-dependent mechanisms [45].

Recent evidence also supports a temporal coupling between HIF-1α activity and cell-cycle progression under normoxic conditions, including a transient G1-phase increase in HIF-1α that promotes survival during nutrient stress [46]. Moreover, oxygen-independent, cell-cycle–dependent expression of HIF-1α under normoxia has been reported in hepatocellular carcinoma cells, where HIF-1α contributes to metabolic preparedness and proliferative competence even under normoxia [47]. Together, these and our findings support a model in which normoxic HIF signaling can modulate both proliferative competence and therapeutic sensitivity in a lineage- and context-dependent manner.

Our results also align with recent work demonstrating that molidustat and roxadustat treatment of hepatoma cells reduced proliferation and enhanced metabolic resilience to mitochondrial inhibition by antimycin A across multiple hepatoblastoma cell lines [48]. Further, the anti-tumor effects observed in our xenograft model are consistent with findings where roxadustat inhibited glioblastoma cell growth by inducing ferroptosis through HIF-2α-mediated upregulation of lipid peroxidation genes including ACSL4 and LPCAT3 [49,50]. However, aforementioned studies did not investigate HIF-deficient models. As a result, all observed effects were attributed to HIF activation rather than HIF-independent mechanisms. Our results extend this framework by revealing that several of these phenotypic effects-particularly altered proliferation, apoptosis resistance, and metabolic remodeling-persist in the absence of HIF-1α and HIF-2α, suggesting additional HIF-independent signaling layers downstream of prolyl hydroxylase inhibition.

In this context, the reduced apoptotic effects observed here and in another study [51] warrant careful interpretation. Transient suppression of apoptosis may support cytoprotection and tissue adaptation under metabolic stress; however, sustained inhibition of programmed cell death could favor survival of genetically unstable cells. Because both HIF activation and PHD inhibition regulate apoptotic mediators such as BCL2, BNIP3L, and survivin, prolonged pharmacological stabilization of HIF pathways might, under certain conditions, influence cell survival dynamics in ways relevant to oncogenesis or therapy resistance. Although meta-analyses of randomized controlled trials in CKD patients [52,53] have not detected increased cancer risk with HIF-PHIs compared to erythropoiesis-stimulating agents, the relatively short study durations (<1 year) may limit detection of delayed oncogenic outcomes. Further, mechanistic and longitudinal studies are needed to clarify how HIF-dependent and HIF-independent apoptotic regulation shapes long-term safety profiles, particularly in patient populations with proliferative or metabolic predispositions.

### 3.3 Metabolic reprogramming: mitochondria, ROS, and lipid metabolism

Our demonstration that roxadustat and molidustat increase mitochondrial membrane potential and reduce ROS independently of HIF-1/2α directly challenges the canonical HIF-1α-driven model in which hypoxia suppresses mitochondrial function and elevates ROS [54,55]. Critically, these drug-induced effects persisted in ΔHIF1ΔEPAS1 cells in which hypoxia itself failed to elicit any response, indicating a HIF-independent mechanism.

Roxadustat-specific, HIF-independent lipid accumulation reveals additional mechanistic complexity beyond the established HIF-2α/ACSL4 ferroptosis pathway [49,50,56]. This observation is consistent with clinical data showing that roxadustat lowers plasma cholesterol and triglycerides in CKD patients, suggesting tissue-specific lipid remodeling that extends beyond erythropoietic effects and may involve direct inhibition of fatty acid oxidation pathways.

To test whether the roxadustat and molidustat modulated transcriptional program in ΔHIF1ΔEPAS1 cells primarily reflects a canonical PHD-loss signature, we compared our most significant 20 up- and down-regulated genes to an independent Egln1/PHD2 deletion signature (GSE77789; GEO2R Top-250). We focused on EGLN1/PHD2 because, among the three EGLN/PHD paralogs, EGLN1 (PHD2) is the primary regulator of HIFα stability, whereas EGLN2/PHD1 and EGLN3/PHD3 generally have more context-dependent or modulatory roles.

The overlap was small (7 genes) and predominantly directionally discordant (5/7 genes change in the opposite direction; Table S3), indicating that the roxadustat/molidustat transcriptional program in HIF-null PC3 cells is not simply explained by PHD2 loss and is consistent with additional regulatory or off-axis mechanisms.

We also sought comparable public “KO vs WT” transcriptome signatures for PHD1/EGLN2 and PHD3/EGLN3 suitable for the same type of comparison, but did not find an equivalent deposited GEO dataset in a comparable format or respective conditions.

It is noteworthy that PHDs have been proposed to regulate additional substrates beyond HIFs [[57], [58], [59]]. While the specificity of individual proposed targets remains debated [60], recent proteomic mapping has substantially strengthened the evidence for broad intracellular proline hydroxylation beyond HIFs [61]. Notably, roxadustat was reported to hydroxylate proteins enriched in RNA metabolism/mRNA splicing and cell-cycle regulation, including mitotic regulators such as Repo-Man/CDCA2 [61]. This resource strengthens the premise that PHD inhibition can alter key cellular programs beyond canonical HIF stabilization.

Importantly, the hydroxylome is a post-translational protein-level dataset, whereas our RNA-seq reflects transcript abundance. Therefore, overlap between these datasets does not distinguish between transcriptional regulation and hydroxylation-dependent changes in protein activity, stability, or localization. Consistent with this distinction, there is limited overlap between strongly induced, hypoxia-responsive transcripts (e.g., CA9, PFKFB4, and BNIP3) and hydroxylated proteins, as would be expected if these genes represented downstream transcriptional outputs rather than primary hydroxylation substrates. However, convergence emerges at the level of core regulatory modules, including RNA processing and cell cycle genes, such as DDX46 and RFC2, which are among our top 20 differentially expressed genes and also appear within hydroxylation-associated pathway intersections.

A mechanistic link between transcript-level reprogramming and protein hydroxylation involves the modification of transcriptional regulators beyond HIF. In line with this, the hydroxylome dataset includes multiple transcription factors, cofactors, and chromatin regulators (e.g., JUN, ATF2, FOXO3, CREBBP, and HDACs). Consistently, within our top 20 RNA-seq lists, JUN itself is induced in the ΔHIF1ΔEPAS1 + roxadustat comparisons. We also observe AP-1–linked downstream outputs, including AREG and CTGF/CCN2, which are relevant to migration and ECM programs. Additionally, FOXO-associated stress/autophagy outputs, such as SESN3 and BNIP3, appear among the top 20 genes. This is consistent with the activation of broader stress response programs, which can influence proliferation and survival phenotypes. Together, these analyses support the interpretation that PHD inhibition can engage broader HIF-independent regulatory networks alongside canonical HIF outputs.

### 3.4 PHD2-dependent, HIF-independent translation regulation

Our transcriptomic data show that roxadustat controls ribosome biogenesis and translation pathways in ΔHIF1ΔEPAS1 cells. This connects to previous studies showing that acute hypoxia or PHD inhibition via DMOG or siRNA rapidly induced translational arrest independent of HIF-α stabilization [62]. Our findings extend this model by showing that roxadustat can trigger a compensatory upregulation of ribosome biogenesis genes, potentially through inhibition of ribosomal oxygenases such as OGFOD1, which hydroxylates Pro-62 in the small ribosomal protein s23 (Rps23) and regulates translation under stress [51,63,64]. The convergence of our transcriptomic findings with mechanistic studies of PHD-dependent translational control underscores the importance of HIF-independent pathways in mediating HIF–PHI biological effects. This pattern indicates a biphasic adaptive process: an initial restriction of translation efficiency followed by a shift toward renewed ribosomal output, supporting cellular resilience during sustained hypoxic or metabolic stress. Such reprogramming of translational control has direct implications for tissue ischemia and tumor contexts, where modulation of ribosome activity critically determines survival and recovery outcomes.

### 3.5 Paradoxical anti-angiogenic effects

Our finding that roxadustat dose-dependently inhibits VEGF-stimulated angiogenesis in HUVECs and suppresses wound healing in both control and ΔHIF1ΔEPAS1 cells contradicts the expected HIF-driven VEGF upregulation by HIF-PHIs. Similar to our findings, the broadly acting HIF-PHD inhibitor DMOG suppresses proliferation, migration, and tube formation in human pulmonary artery endothelial cells [65]. One potential explanation is off-target CP4H inhibition, where increased 2-OG may enhance PHD2-mediated HIF-1α degradation [66]. Alternatively, unfavorable metabolic reprogramming impairing angiogenic competence, differential effects on inflammatory cytokines and oxidative stress, interference with receptor tyrosine kinase signaling, or changes in extracellular matrix remodeling could contribute to the net anti-angiogenic effects. Clinically, this raises concerns in CKD patients on chronic HIF–PHI therapy. Although meta-analyses to date have found no overall increased cardiovascular risk with HIF-PHIs, the WHITNEY trial reported elevated DVT (15.2%) and PE (9.8%) with roxadustat, indicating context-specific thrombotic risks [67] that warrant further investigations.

### 3.6 Clinical implications and repurposing opportunities

HIF-PHIs such as roxadustat and molidustat are established treatments for anemia in CKD, effectively raising hemoglobin and improving iron metabolism with safety profiles largely comparable to ESAs and placebo, though serious adverse events, notably thromboembolic risks, require ongoing vigilance [52,53,[67], [68], [69]].

Our demonstration of robust HIF-independent effects, including metabolic reprogramming and anti-tumor activity, suggests promising therapeutic potential in oncology and metabolic diseases, especially where modulation of cellular stress pathways or hypoxia resilience is beneficial [[48], [49], [50]].

Ongoing and planned clinical trials in myelodysplastic syndromes and non-renal anemia indications further support the feasibility of repurposing these agents. Nonetheless, extended follow-up is critical to fully characterize the long-term risk–benefit balance, particularly regarding cancer, thrombosis, and tissue repair in diverse patient populations.

Altogether, our findings illuminate mechanisms underlying both potential adverse effects in CKD patients and alternative therapeutic pathways for broader clinical application, establishing a framework for optimizing HIF–PHI safety and efficacy through structure-guided design and long-term clinical monitoring.

## 4 Limitation of the study

A limitation of the study is that, while we demonstrate that PHD inhibitors elicit biological and transcriptional responses beyond canonical HIF-1α/HIF-2α stabilization, we do not dissect the ultimate molecular mechanisms driving these HIF-independent effects. Our work was designed to establish the existence of these additional pathways using functional assays and unbiased RNAseq profiling, but not to determine which specific hydroxylases, substrates, or 2OGDD family members mediate them. Consequently, potential contributions from individual PHD isoforms, FIH, non-HIF substrates remain to be experimentally defined. Likewise, differences in target selectivity between roxadustat and molidustat were not explored at the biochemical level. These mechanistic questions represent important avenues for future research but were beyond the scope of the present study, which aimed primarily to reveal that clinically used PHD inhibitors exert broader, non-canonical cellular effects.

## 5 Materials and methods

All biochemicals and enzymes were of analytical grade and were obtained from commercial suppliers. Roxadustat was purchased from Cayman chemicals and molidustat was puchased from Selleckchem. For cell experiments, all substances were dissolved in DMSO.

### 5.1 Cell culture

Human prostate cancer cells (PC-3, #CRL-1435) and HEK-293 cells (#CRL-1573) were authenticated and purchased from ATCC. PC-3 cells were cultured under normoxia (16% O_2_, 79% N_2_, and 5% CO_2_ [v/v]) in RPMI 1640 medium supplemented with 10% FBS. HEK-293 cells were maintained in MEM. Both cell lines were tested mycoplasma negative by using the MycoAlert Detection Kit (Lonza) and in all experiments the number of cell passages used was below 10. For RNA and protein extraction, cells were seeded onto 60 mm dishes. After a medium change, cells were treated with roxadustat (10 μM) or molidustat (5 μM), further cultured for 16 h either under normoxia or hypoxia (5% O_2_, 90% N_2_, and 5% CO_2_ [v/v]) and then harvested. In all experiments control cells were treated with DMSO.

### 5.2 Generation of HIF-1α and HIF-2α double knockout PC-3 cells by CRISPR/Cas9-mediated genome editing

Two 20-bp guide sequences targeting the second exon human HIF-1α (HIF-1α-201, HIF-1α-202, HIF-1α-203, HIF-1α-204, ENSG00000100644) or the second exon human HIF-2α (EPAS1-201, EPAS1-202, ENSG00000116016) were designed online using Zhang's laboratory web resource (www.genome-engineering.org); a non-targeting, scrambled sequence (OriGene) was used as a negative control. gRNA-encoding oligonucleotides (Sigma-Aldrich) were cloned into the vector SpCas9(BB)-2A-GFP (PX458, Addgene plasmid #48138) using standard procedures as described [70]. The generation of the control and double-knockout cells via CRISPR/Cas9-mediated non-homologous end-joining (NHEJ) DNA repair and the screening was performed according to described guidelines [70]. In brief, the wild-type PC-3 cells were transiently transfected with either the genome editing constructs (herein referred to DHIFDEPAS) or the scrambled CRISPR/Cas9 construct (herein referred to control Ctl) and 48 h post-transfection cells were subjected to single-cell-sorting (BD FACSAria™ III cell sorter). The single-cell clones were expanded and screened for frame-shift mutations; shortly, a region spanning the target site was amplified by PCR from genomic DNA isolated from clonal cell lines. PCR products were subsequently cloned into pUC19 (Invitrogen). 15-20 sequences were analyzed per clone by aligning them to the WT *HIF1A* or WT *EPAS1* sequences using BLAST and Serial Cloner. All primer sequences are listed in Supplementary Table S4.

### 5.3 Western blot analysis

Western blot analysis was carried out as previously described [71]. In brief, lysates from PC-3 cells were collected, and 50-100 μg of protein was loaded onto a 7.5% or 10% sodium dodecyl sulfate (SDS)- polyacrylamide gel. After electrophoresis and electroblotting onto a nitrocellulose membrane, proteins were detected with antibodies listed in Supplementary Table S5. The ECL system (Amersham) was used for detection. Blots of three independent experiments were quantified by densitometry with the Image Quant TL Program (GE Healthcare); densitometry data were normalized to α-tubulin.

### 5.4 Plasmid constructs and luciferase assay

The plasmid construct for pGL3-EPO-HRE-Luc has been previously described [72]. 4 x 10^5^ cells per 6 cm dish were transfected as previously described [73]. In brief, cells were cotransfected with 2.5 μg of pGL3-EPO-HRE-Luc and 0.25 μg Renilla luciferase construct (pRLSV40, Promega) for normalization. After 24 h the medium was changed, cells were treated with roxadustat (10 μM) or molidustat (5 μM) and further cultured for additional 24 h. The detection of luciferase activity was performed with the Dual - Luciferase™ Reporter Gene Assay Kit (Berthold, Pforzheim, Germany).

### 5.5 Cell viability

Cell viability was assessed by a MTT [3-(4,5-dimethylthiazol-2-yl)-2,5-diphenyl tetrazolium bromide] reduction assay. PC-3 cells were seeded in 24-well plates (4 x 10^4^ cells per well) and cultured under normoxia (16% O_2_). After 24 h cells were treated with either roxadustat (10 μM) or molidustat (5 μM) and further cultured for 24 h under normoxic conditions (16% O_2_). Then 500 μl of MTT (4 mg/ml) was added to the wells and incubated at 37 °C for 1 h. Formation of MTT–formazane was measured with a Tecan plate reader at a wavelength of 570 nm.

### 5.6 Colony formation assay, BrdU incorporation and adhesion assay

For colony formation, PC-3 cells were plated into 6-well plates at a density of 2.000 cells/well and allowed to seed for 6 h before inhibitor treatment (roxadustat: 10 μM; molidustat: 5 μM). Every second day, medium was replaced with fresh inhibitors. After 10 days, cells were fixed with 4% paraformaldehyde (PFA) and stained with crystal violet (Applichem). Plates were scanned and analyzed using ImageQuant TL (Colony version 7.0 (GE Healthcare)).

For BrdU incorporation cells were seeded onto 96-well plates at a density of 10.000 cells per well and allowed to settle overnight. After a medium change, cells were treated with either roxadustat (10 μM) or molidustat (5 μM) and labeled with bromodeoxyuridine (BrdU) for 24 h. The BrdU cell proliferation kit (Calbiochem/Merck) was used for detection.

For adhesion assay, cells were seeded onto 6-well plates, cultured until they reached confluency and then treated with roxadustat (10 μM) or molidustat (5 μM) for 24 h. Thereafter, cells were transferred to an orbital shaker and rotated at 500 rpm for 8 h at 37 °C. Control plates remained in the incubator. After rotation, cells were washed twice with 1x PBS, fixed with 4% PFA for 20 min at room temperature and stained with crystal violet solution. Subsequently, the cells were rinsed with water three times and air dried. Plates were scanned and analyzed using Fiji.

### 5.7 Analysis of cell cycle and cell death

Cell cycle and cell apoptosis were measured in synchronized cells by Annexin-V-FITC and propidium iodide (PI) staining kits (MedChemExpress) according to the manufacturer's protocol. For synchronization, confluent cells were kept without medium change for 24 h. Release into the cell cycle was achieved by subculture and incubation with fresh serum with or without roxadustat (10 μM) or molidustat (5 μM) for indicated time points. Apoptosis and cell cycle parameters were analyzed using a BD LSR-Fortessa (Becton Dickinson) flow cytometer.

### 5.8 High-throughput screening of mitochondria membrane potential, reactive oxygen species (ROS), acidic organelles and neutral lipids

PC-3 control (Ctl) and PC-3 DHIFDEPAS cells were grown on 96-well plates and treated with either roxadustat (10 μM) or molidustat (5 μM) for 24 h. Thereafter, cells were stained with either 200 nM tetramethylrhodamine TMRE (ab113852, abcam), 5 μM CellROX® (C10422, Invitrogen), 75 nM LysoTracker™ (L12492, Invitrogen) or 1x HCS LipidTOX™ (H34475, Invitrogen) for 30 min. Cells were analyzed with the Operetta high-content system (PerkinElmer) by exciting the fluorophores with the respective wavelengths (emission/excitation: 549/575 nm for TMRE, 644/665 nm for CellROX®, 647/668 for LysoTracker™ and 495/505 nm for LipidTOX™). Quantification of the measurements were performed using the Operetta software “Harmony” (PerkinElmer).

### 5.9 Measurement of ROS with H_2_DCF-DA

Measuring ROS was performed on a 96-well plate by using the non-fluorescent dye dichlorodihydrofluorescein diacetate (H_2_DCF-DA). Cells were treated with roxadustat (10 μM), molidustat (5 μM) and Mn(III)TBAP (20 μM) for 24 h and then stimulated with H_2_O_2_ (500 μM) for additional 30 min. Thereafter, cells were washed with 1x PBS and 10 μM H_2_DCF-DA was added for 30 min at 37 °C in dark. Fluorescence in each well was measured with a Tecan fluorescence plate reader at 37 °C with suitable wavelenght for DCF (Ex 495 nm, Em 529 nm).

### 5.10 Live cell imaging assays

For real-time quantitative live-cell proliferation assay, PC-3 cells were seeded onto 96-well plates (3x10^3^ cells/well) in full growth media. After a medium change on the following day, the cells were treated with either roxadustat (10 μM) or molidustat (5 μM) and the plate was moved into the InCucyte®S3 (Sartorius) for live phase contrast imaging for 72 h in 3 h intervals. For scratch wound assay, PC-3 cells were seeded onto 96-well Incucyte®Imagelock Plates (Sartorius) (1.5 x 10^4^ cells/well) in RPMI1640 media supplemented with 10% FBS. The following day the confluent cell monolayer was wounded with the 96 PTFE pin Incucyte®Woundmaker (Sartorius), washed twice with 1x PBS, and further cultured in RPMI1640 supplemented with 0.1% FBS and either roxadustat (10 μM) or molidustat (5 μM). The live wound closure was phase contrast-imaged for 72 h in 3 h intervals and the confluence analysis was performed using the InCucyte®Scratch Wound Analysis software.

### 5.11 Angiogenesis fibrin bead assay

Human umbilical vein endothelial cells (HUVECs) were isolated following a previously established protocol [74], using 3 mg per 10 ml concentration collagenase. The cells were cultured in PromoCell Endothelial Cell Growth Medium (Heidelberg, Germany), supplemented with a 1x endothelial cell growth kit and 10% FBS. Experiments were conducted using HUVECs at passage 2. Human primary lung fibroblasts (hPFs), obtained from PromoCell, were cultured in DMEM containing 10% FBS and used at passage 9. Collection of umbilical cords for cell isolation was approved by Ethics Committee of the Kuopio University Hospital (Kuopio, Finland, 341/2015).

3D in vitro model mimicking angiogenesis was performed as described previously [75,76]. Briefly, HUVECs (passage 2) were seeded on top of collagen-coated Cytodex 3 beads (GE Healthcare, Little Chalfont, UK) and cultured in a fibrin gel (2.5 mg/ml fibrinogen in DPBS, 0.15 U/ml aprotinin, 3 μl (10 U/ml) per well thrombin; Merck KGaA, Darmstadt, Germany). Fibroblasts (10.000 cells/well) and compound stimulations were replaced every other day during 3-7 days follow up. After fixation, the cells were labeled with Phalloidin-AG35 (Thermo Fisher Scientific) and DAPI. Images were taken with a confocal laser scanning microscope (Zeiss LSM800): 405/555 nm diode lasers were used together with the appropriate emission filters (10 x/0.3 PlanApo objective, 1024× 1024 frame size). Image processing and analysis was performed using Fiji [77].

### 5.12 *In vivo* xenograft mouse model

1x10^6^ PC-3 control (Ctl) and PC-3 DHIFDEPAS knockout cells were injected subcutaneously into both flanks (two spots per flank) of 12 female athymic nude mice (Envigo). 5 days after cell injection, mice received three weekly intraperitoneal (i.p.) injections of roxadustat (20 mg/kg body weight in 5% DMSO, 5% Tween-80, and 90% physiological saline) over a 4-week period. Control mice were treated with the solvent without adding roxadustat. The animals were housed at the Laboratory Animal Center of the University of Oulu in IVC cages with a 12-h light/dark cycle and food and water *ad libitum*. All the experiments were performed according to protocols approved by the National Animal Experiment Board of Finland (ELLA).

### 5.13 RNA seq

PC-3 control (Ctl) and PC-3 ΔHIF1ΔEPAS1 cells were seeded in 60 mm dishes one day prior to treatments with either DMSO, hypoxia (5% O_2_), 10 μM roxadustat or 5 μM molidustat for 16 h. RNA was extracted using the RNAeasy Mini Kit (Qiagen; 74104) according to manufacturer's protocol. Genomic DNA was removed from RNA samples using RNase-free DNase (Qiagen; 79254). RNA quality evaluation (yield, purity and integrity), cDNA library construction, Illumina sequencing as well as bioinformatic analysis were performed by Novogene (Oxford, United Kingdom).

Raw reads of fastq format were firstly processed through in-house perl scripts. In this step, clean data (clean reads) were obtained by removing reads containing adapter, reads containing ploy-N, and low-quality reads from raw data.29 All the downstream analyses were based on the clean data with high quality. Reference genome (GRCh37/hg19) and gene model annotation files were downloaded from genome website directly. Index of the reference genome was built using Hisat2 v2.0.5 and paired-end clean reads were aligned to the reference genome using Hisat2 v2.0.5. Then, the abundance of each transcript was quantified using feature Counts v1.5.0-p3. Differentially expressed genes (DEGs) analysis was performed using the DEseq2 package, and gene expression was normalized using the relative-log-expression (RLE) in DESeq2.30 Genes with an adjusted P-value <0.05 found by DESeq2 were assigned as differentially expressed. For gene ontology (GO) analysis, metascape (http://metascape.org) was employed.

### 5.14 Statistical analysis

Densitometry data were plotted as fold induction of relative density units, with the zero-value absorbance in each figure set arbitrarily to 1 or 100%. Prism 10 software (GraphPad; Version 10.2.2) was used to perform statistical analysis. Two-tailed Student's *t*-test was used to test differences between two groups and differences between multiple groups were analyzed by ordinary one-way ANOVA. Data are shown as mean ± SD of three biological replicates. For all statistical tests, *p* values < 0.05 were considered significant. All experiments were repeated at least three times with similar results.

## Material availability

All unique/stable reagents generated in this study are available from the corresponding authors with a completed materials transfer agreement. Requests should be submitted to: Daniela Mennerich (daniela.mennerich@oulu.fi) or Thomas Kietzmann (thomas.kietzmann@oulu.fi)

## Funding

This work was supported by the Research Council of Finland (TK SA356920), the Jane and Aatos Erkko Foundation (TK 210031), the Finnish Cancer Foundation, the Sigrid Juselius Foundation, the RCF Flagship Program GeneCellNano (SYH SA374293), the University of Oulu, and Biocenter Oulu as a member of Biocenter Finland.

## CRediT authorship contribution statement

**Daniela Mennerich:** Conceptualization, Investigation, Writing – original draft, Writing – review & editing. **Fawzi Khoder-Agha:** Investigation, Writing – review & editing. **Mustafa Beter:** Investigation, Writing – review & editing. **Elitsa Y. Dimova:** Investigation. **Seppo Ylä-Herttuala:** Funding acquisition, Resources, Writing – review & editing. **Thomas Kietzmann:** Conceptualization, Funding acquisition, Investigation, Resources, Writing – original draft, Writing – review & editing.

## Declaration of competing interest

The authors declare that they have no known competing financial interests or personal relationships that could have appeared to influence the work reported in this paper.

## Data Availability

All data needed to evaluate the conclusion in the paper are present in the paper and/or the Supplementary Data. The RNAseq data generated in this study will be deposited in the NCBI Gene Expression Omnibus (GEO) database and made publicly available upon publication under the accession number GSE328627. Any additional information required to reanalyze the data reported in the paper is available from the corresponding authors upon request.
