# Supplementary material for: Clinically approved HIF-PHIs modulate redox metabolism, cell growth, and angiogenesis independent of HIF-1α/HIF-2α
Source: Redox Biol. 2026 May 14;94:104206. doi: 10.1016/j.redox.2026.104206 (PMC13208096; doi:10.1016/j.redox.2026.104206)
Supplement: Multimedia component 1 [file mmc1.docx]

**Supplementary material for**

**Clinically approved HIF-PHIs modulate redox metabolism, cell growth, and angiogenesis independent of HIF-1α/HIF-2α**

Daniela Mennerich^1^*, Fawzi Khoder-Agha^1^, Mustafa Beter^2^, Elitsa Y. Dimova^1^, Seppo Ylä-Herttuala^2^ and Thomas Kietzmann^1^*

^1^Faculty of Biochemistry and Molecular Medicine, and Biocenter Oulu, University of Oulu, FI-90014 University of Oulu, Finland

^2^ A.I. Virtanen Institute for Molecular Sciences, University of Eastern Finland, FI-70211 Kuopio, Finland

*Corresponding authors

**Contents**

**Figure S1. High concentrations of roxadustat and molidustat inhibit cell viability.**

**Figure S2. Roxadustat and molidustat induce HIF-target gene expression as well as HRE-Luc activity.**

**Figure S3. High concentrations of roxadustat and molidustat inhibit cell proliferation.**

**Figure S4. Roxadustat and molidustat do not affect cell adhesion.**

**Figure S5. Roxadustat and molidustat inhibit proliferation and colony formation in the absence of HIF-PHD1-3.**

**Figure S6: Roxadustat and molidustat do not increase reactive oxygen species.**

**Figure S7. Roxadustat affects angiogenesis in HUVEC cells.**

**Figure S8. Correlation of the RNA-seq samples.**

**Figure S9. Venn diagrams of all different treatment groups.**

**Figure S10. The 20 most significant up- and downregulated genes in ΔHIF1ΔEPAS1 cells treated with roxadustat.**

**Figure S11. The 20 most significant up- and downregulated genes in ΔHIF1ΔEPAS1 cells treated with molidustat.**

**Table S1: List of the 20 most significant up- and downregulated genes in ΔHIF1ΔEPAS1 cells treated with roxadustat.**

**Table S2: List of the 20 most significant up- and downregulated genes in ΔHIF1ΔEPAS1 cells treated with molidustat.**

**Table S3: Overlap between our Top‑20 lists and GSE77789 (Egln1/PHD2 KO) Top‑250 (GEO2R)**

**Table S4: Nucleotide sequences used for CRISPR-Cas9 gene editing.**

**Table S5: List of antibodies used in the study.**

**
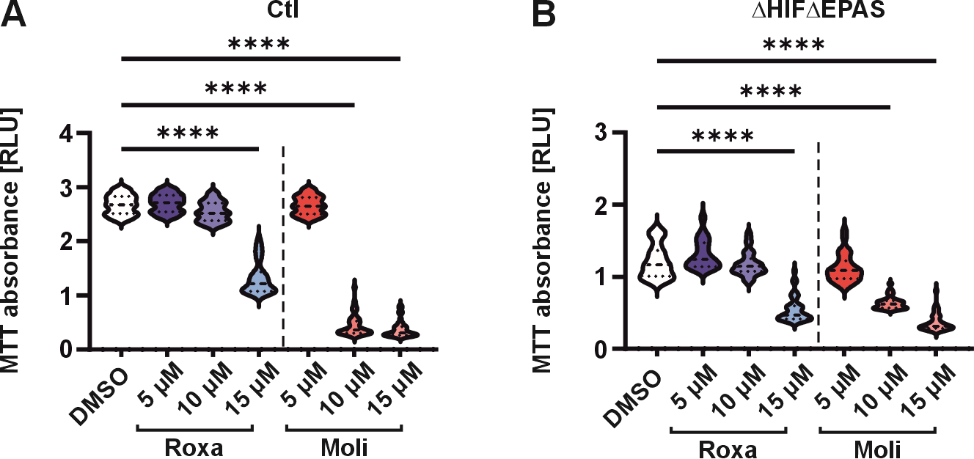
**

**Figure S1. High concentrations of roxadustat and molidustat inhibit cell viability.**

**(A,B)** PC-3 control (Ctl) and ΔHIF1ΔEPAS1 cells were cultured for 24 h, then treated with roxadustat (Roxa) or molidustat (Moli) at different concentrations and further cultured for 24 h. Thereafter, an MTT assay was performed.

**
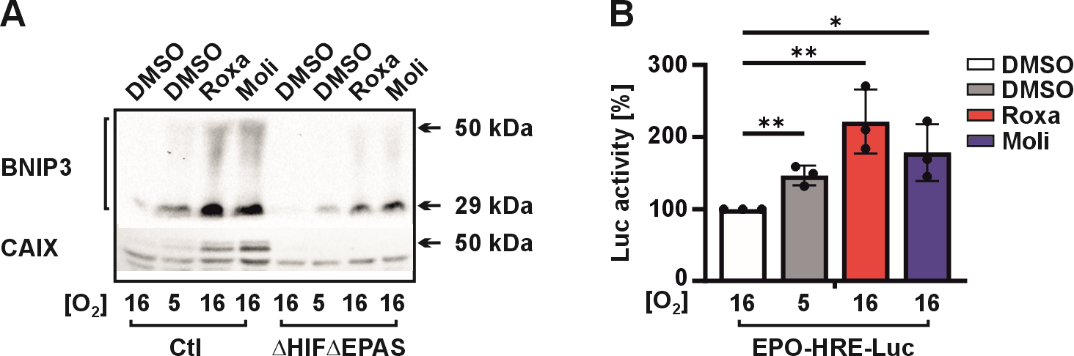
**

**Figure S2. Roxadustat and molidustat induce HIF-target gene expression as well as HRE-Luc activity.**

**(A)** PC-3 control (Ctl and ΔHIF1ΔEPAS1 cells were cultured for 24 h, then treated with either roxadustat (Roxa) or molidustat (Moli) and further cultured under normoxia or hypoxia (5%O_2_) for 16 h. Total protein levels were measured by Western blot with antibodies against BNIP3, CAIX and α-tubulin. **(B)** HEK 293 cells were transfected with an EPO-HRE-Luc expression plasmid for 5 h. After a medium change, cells were treated with roxadustat (Roxa) or molidustat (Moli) and further cultured under normoxic or hypoxic (5%O_2_) conditions for 24 h.

**
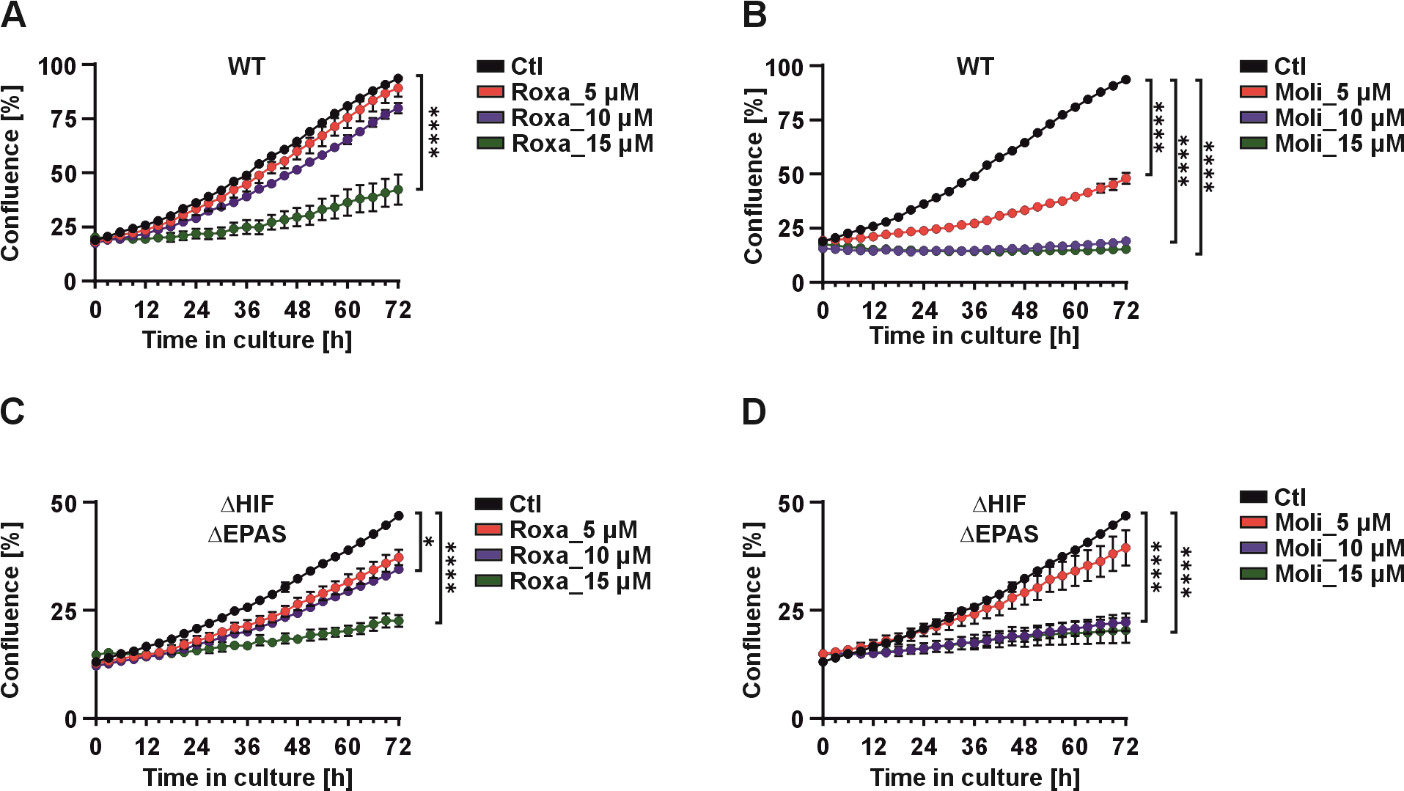
**

**Figure S3. High concentrations of roxadustat and molidustat inhibit cell proliferation.**

**(A - D**) PC-3 control (Ctl) and ΔHIF1ΔEPAS1 cells were cultured for 16 h, after which they were treated with roxadustat (Roxa) or molidustat (Moli) at different concentrations. The live cell proliferation rate was measured with the InCucyte®ZOOM every 3 h for 72 h.

**
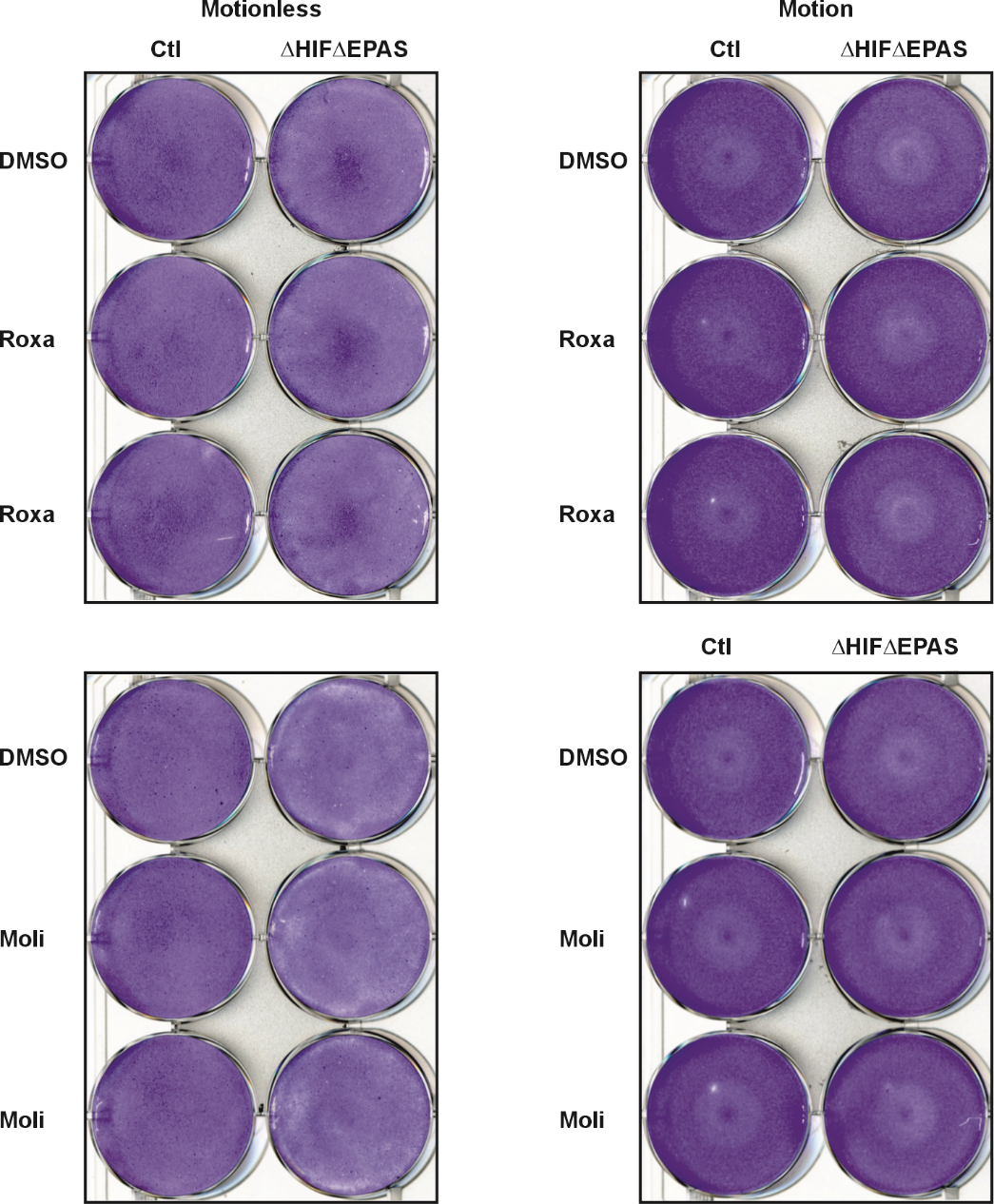
**

**Figure S4. Roxadustat and molidustat do not affect cell adhesion.**

PC-3 control (Ctl) and ΔHIF1ΔEPAS1 cells were plated on 6-well plates and grown until confluence was reached. The cells were then treated with roxadustat (Roxa; 10 µM) or molidustat (Moli; 5 µM) and further cultured for 24 h. Thereafter, the plates were transferred to an orbital shaker for an additional 8 h. Control plates remained in the cell incubator.


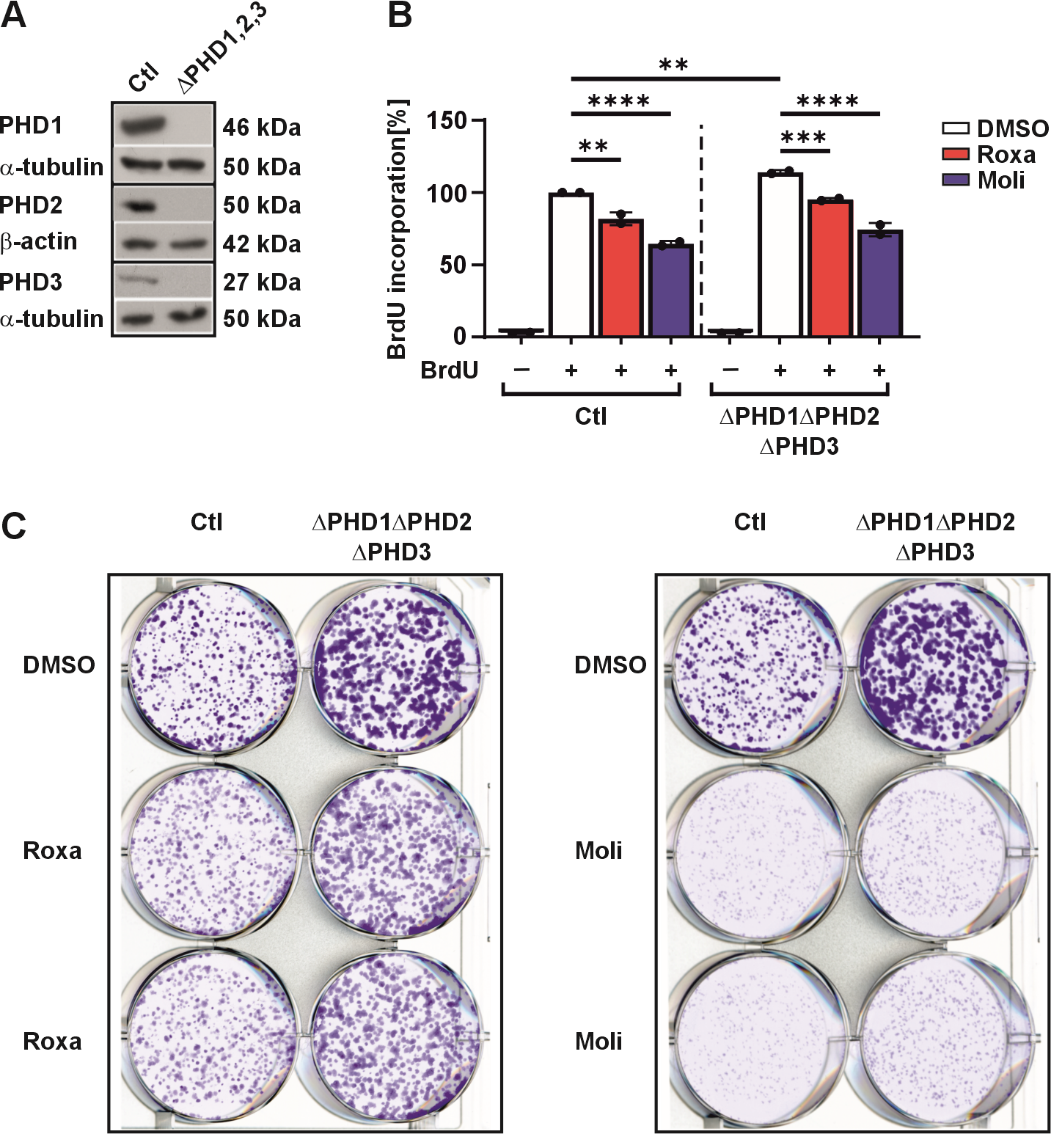


**Figure S5. Roxadustat and molidustat inhibit proliferation and colony formation in the absence of HIF-PHD1-3.**

**(A)** Representative Western blot analysis. 100 µg of total protein lysate from control B16-F10 mouse melanoma cells and ΔPHD1,2,3 cells was analyzed with antibodies against PHD1, PHD2, PHD3 and α-tubulin. **(B)** BrdU incorporation. Cells were treated with roxadustat (Roxa; 10 µM) or molidustat (Moli; 5 µM) for 24 h and then further analyed. Statistics: Ordinary one-way ANOVA. *p<0.05, **p<0.01, ***p<0.001 ****p<0.0001. **(C)** Colony formation. Representative images of stained colonies fromcontrol (Ctl) and ΔPHD1,2,3 cells. Cells were seeded onto 6-well plates at a density of 2,000 cells/well, allowed to settle for 24 h, and then treated with roxadustat (Roxa; 10 µM) or molidustat (Moli; 5 µM). The cells were cultured for an additional 8 days. Every second day, medium was replaced with fresh inhibitors. Afterwards, cells were fixed with 4% paraformaldehyde and stained with crystal violet.

**
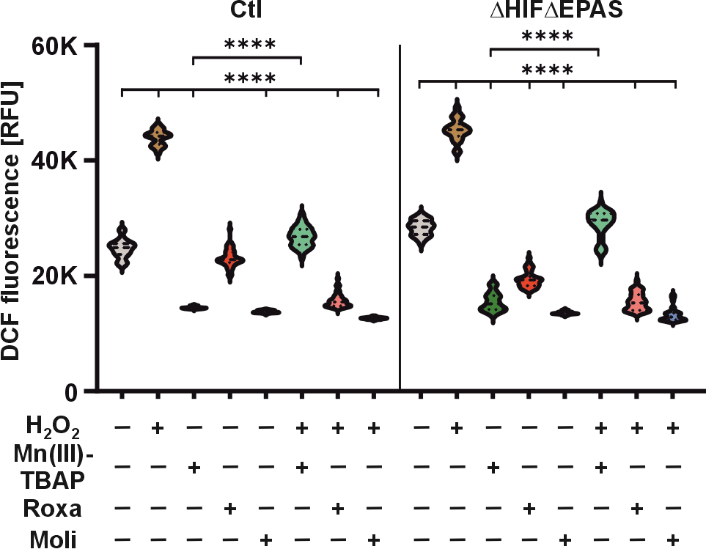
**

**Figure S6. Roxadustat and molidustat do not increase reactive oxygen species.**

PC-3 control cells (Ctl) and ΔHIF1ΔEPAS1 cells were treated with roxadustat (10 µM), molidustat (5 µM) or Mn(III)TBAP (20 µM) for 24 h and then stimulated with H_2_O_2_ (500 µM) for additional 30 min.

**
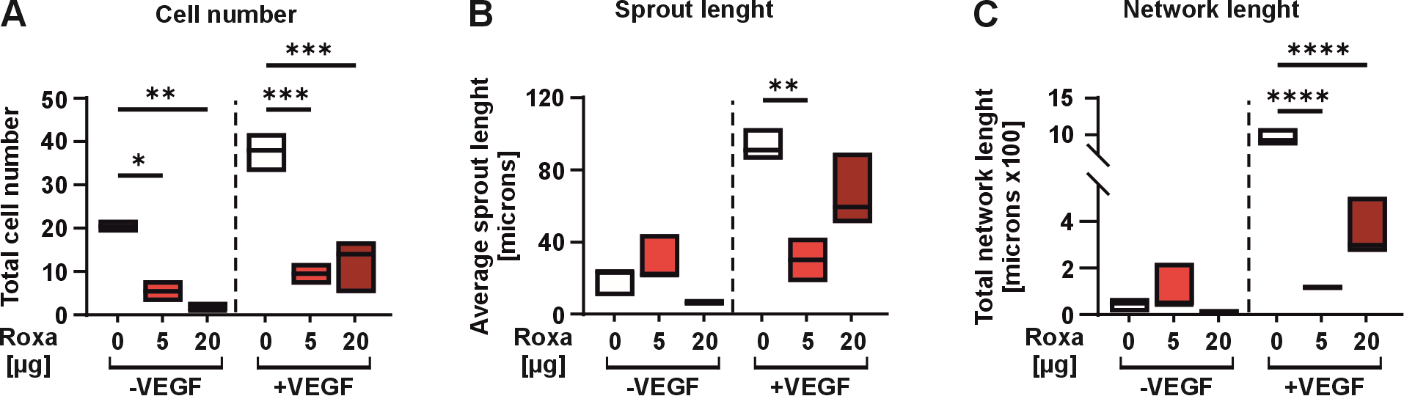
**

**Figure S7. Roxadustat affects angiogenesis in HUVEC cells.**

Quantification of the angiogenesis assay in HUVEC cells after 3 days of roxadustat (Roxa) treatment for 3 days: **(A)** cell number, **(B)** sprout length, and **(C)** network length.

**
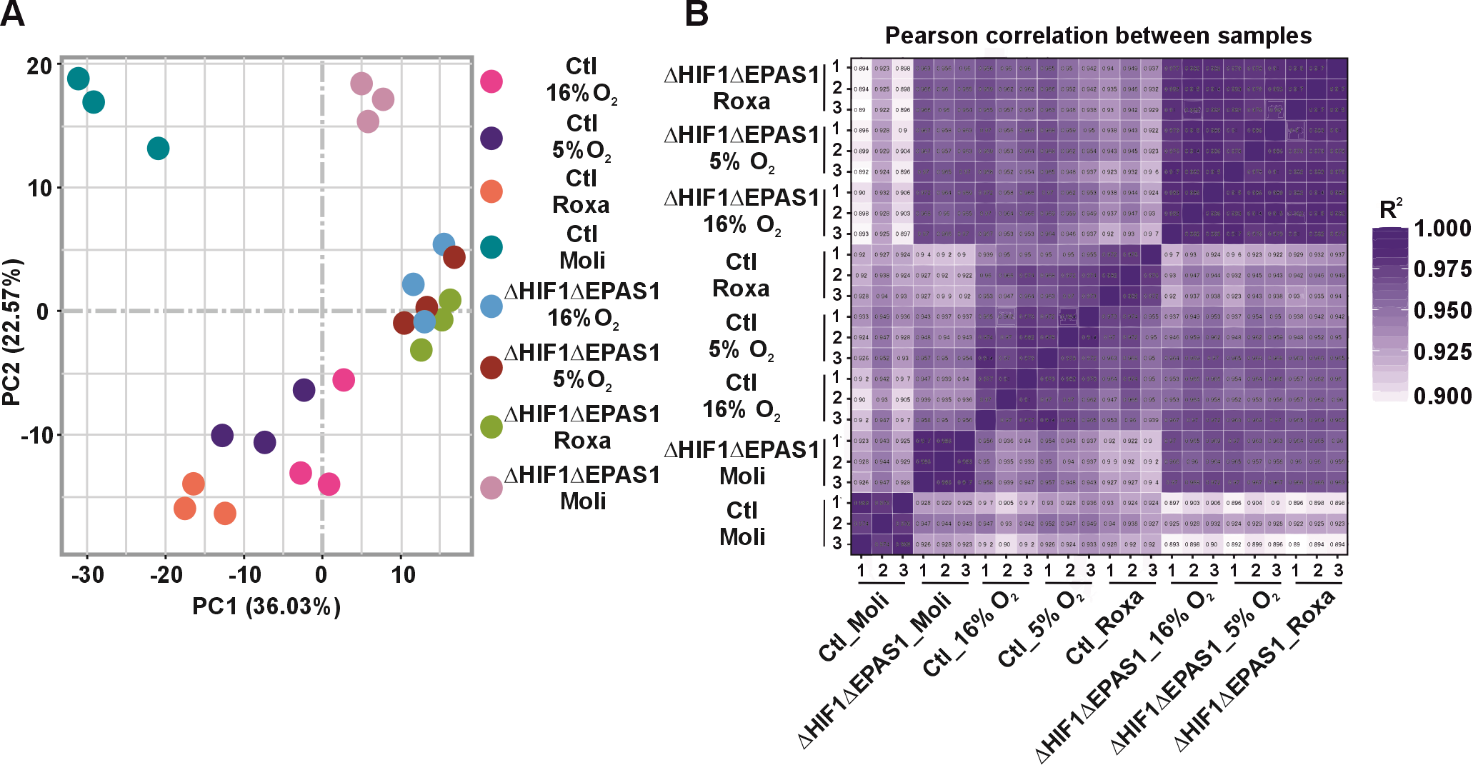
**

**Figure S8. Correlation of the RNA-seq samples.**

**(A)** Principal component analysis (PCA) was performed on normalized gene expression data from all samples. Each point represents an individual sample and is colored by experimental group. **(B)** Pearson correlation between the samples.

**
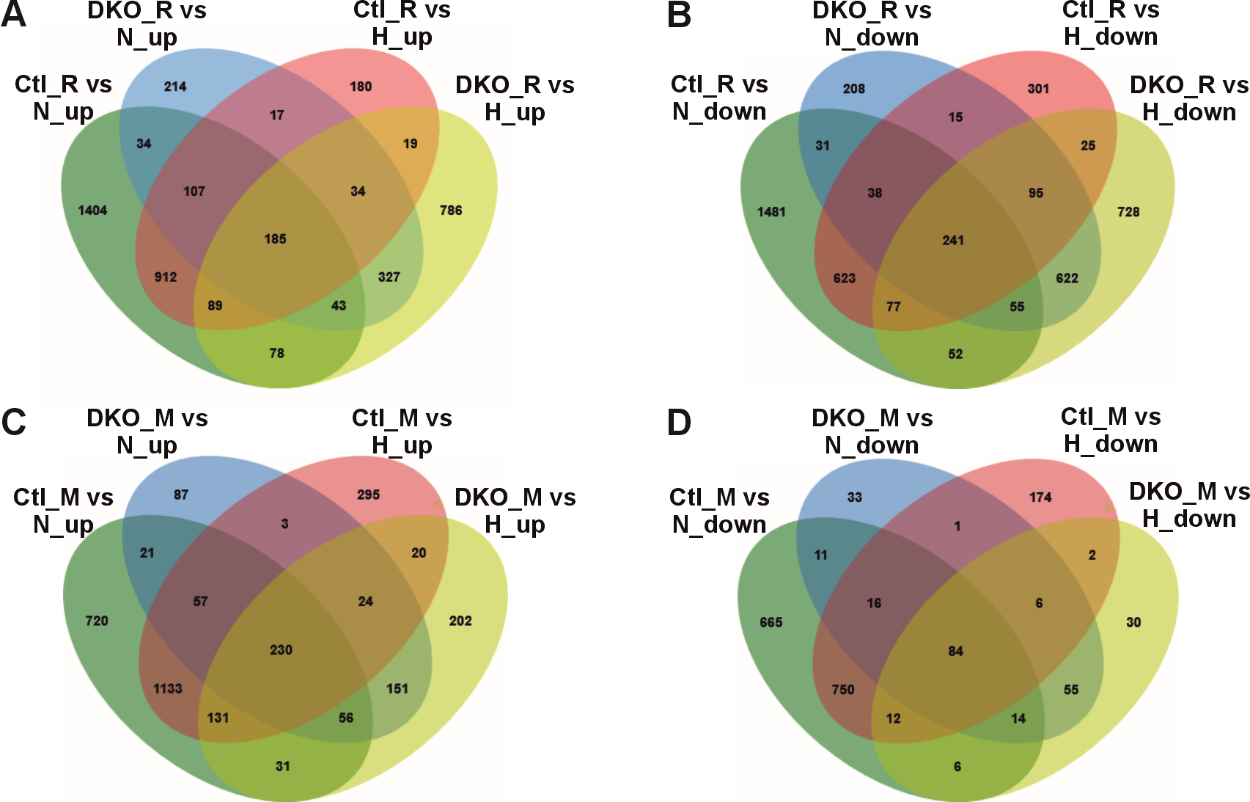
**

**Figure S9. Venn diagrams of all different treatment groups.**

Venn diagrams of differentially expressed unique and shared genes. **(A)** Upregulated genes in control (Ctl) or ΔHIF1ΔEPAS1 (DKO) cells upon roxadustat (R) treatment. **(B)** Down-regulated genes in control (Ctl) or ΔHIF1ΔEPAS1 (DKO) cells upon roxadustat (R) treatment. **(C)** Upregulated genes in control (Ctl) or ΔHIF1ΔEPAS1 (DKO) cells upon molidustat (M) treatment. **(D)** Down-regulated genes in control (Ctl) or ΔHIF1ΔEPAS1 (DKO) cells upon molidustat (M) treatment.

**
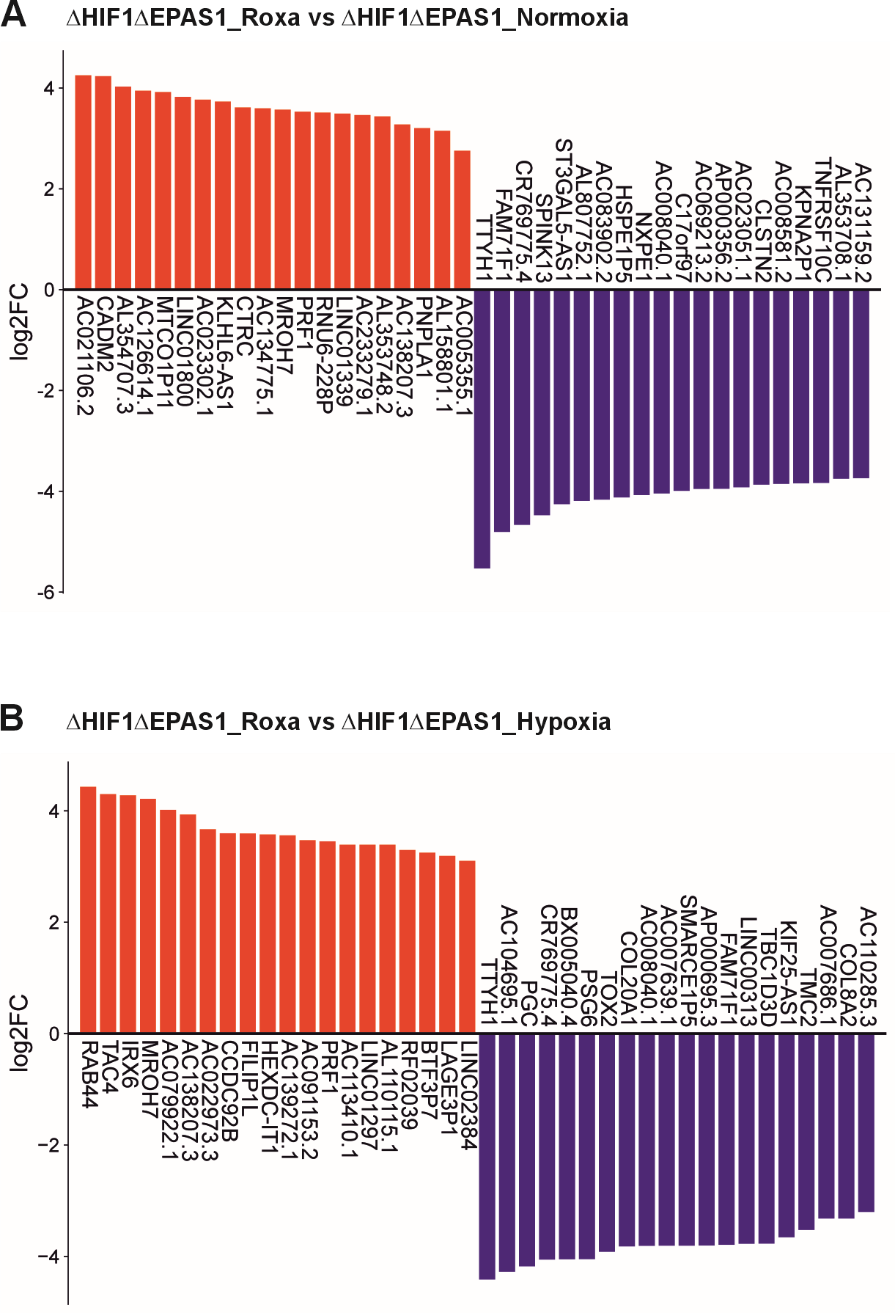
**

**Figure S10. The 20 most significant up- and downregulated genes in ΔHIF1ΔEPAS1 cells treated with roxadustat.**

**(A**) Most up- and downregulated genes in ΔHIF1ΔEPAS1 cells upon roxadustat (Roxa) treatment compared to normoxia. **(B)** Most up- and downregulated genes in ΔHIF1ΔEPAS1 cells upon roxadustat (Roxa) treatment compared to hypoxic conditions.

**Table S1: List of the 20 most significant up- and downregulated genes in ΔHIF1ΔEPAS1 cells treated with roxadustat.**

| **ΔHIF1ΔEPAS1_Roxa vs ΔHIF1ΔEPAS1_16%_up** | |  |  |
| --- | --- | --- | --- |
|  |  |  |  |
| **gene_name** | **gene description** | **log2FoldChange** | **pvalue** |
| AC021106.2 | ‒ | 4.25110381425737 | 0.016369509169116 |
| CADM2 | cell adhesion molecule 2 | 4.23204212257094 | 0.0162207699951032 |
| AL354707.3 | ‒ | 4.02697442966771 | 0.0327610238484835 |
| AC126614.1 | ‒ | 3.94489010475098 | 0.04011068455859 |
| MTCO1P11 | mitochondrially encoded cytochrome c oxidase I pseudogene 11 | 3.91983939809433 | 0.0389054231138303 |
| LINC01800 | long intergenic non-protein coding RNA 1800 | 3.82175636674758 | 0.0251366658568029 |
| AC023302.1 | ‒ | 3.76782241215689 | 0.0187779865741235 |
| KLHL6-AS1 | KLHL6 antisense RNA 1 | 3.72842647184467 | 0.0339741141919712 |
| CTRC | chymotrypsin C | 3.61412422484737 | 0.0313237266541947 |
| AC134775.1 | ‒ | 3.59698787836515 | 0.0295978126506214 |
| MROH7 | maestro heat like repeat family member 7 | 3.57010332518439 | 0.0495947775303425 |
| ANGPTL4 | angiopoietin like 4 | 3.54327893702169 | 5.53857948504564e-21 |
| PRF1 | perforin 1 | 3.52801224352852 | 0.03762723941479 |
| RNU6-228P | RNA, U6 small nuclear 228 | 3.51016794047536 | 0.0345519966594117 |
| LINC01339 | long intergenic non-protein coding RNA 1339 | 3.49162869214271 | 0.0407609520573268 |
| AC233279.1 | ‒ | 3.46175590753035 | 0.0179148842845204 |
| AL353748.2 | ‒ | 3.43719869634609 | 0.0189052802875152 |
| AC138207.3 | ‒ | 3.275161739182 | 0.0352898234156878 |
| PNPLA1 | patatin like phospholipase domain containing 1 | 3.20274554041267 | 0.036139677790327 |
| AL158801.1 | ‒ | 3.15041182047568 | 0.0351164590093427 |
|  |  |  |  |
|  |  |  |  |
| **ΔHIF1ΔEPAS1_Roxa vs ΔHIF1ΔEPAS1_16%_down** | |  |  |
|  |  |  |  |
| **gene_name** | **gene_description** | **log2FoldChange** | **pvalue** |
| TTYH1 | tweety family member 1 | -5.5305139472315 | 0.0049239409759158 |
| FAM71F1 | family with sequence similarity 71 member F1 | -4.8069104981950 | 0.0021845410308173 |
| CR769775.4 | ‒ | -4.6653508918063 | 0.0031762556817239 |
| SPINK13 | serine peptidase inhibitor, Kazal type 13 | -4.4733064495421 | 0.0094344366116633 |
| AC019077.1 | ‒ | -4.3299906874013 | 0.0088265931160631 |
| ST3GAL5-AS1 | ST3GAL5 antisense RNA 1 | -4.2563241566824 | 0.0134075878812257 |
| AL807752.1 | ‒ | -4.1927824416610 | 0.0131840341460607 |
| AC083902.2 | Homo sapiens uncharacterized mRNA | -4.1653022604615 | 0.0213993916612154 |
| HSPE1P5 | heat shock protein family E (Hsp10) member 1 pseudogene 5 | -4.1214163951244 | 0.0198136221176007 |
| NXPE1 | neurexophilin and PC-esterase domain family member 1 | -4.0758021942240 | 0.0180843190710995 |
| AC008040.1 | ‒ | -4.0418256716972 | 0.025958513992419 |
| C17orf97 | chromosome 17 open reading frame 97 | -3.9915530511858 | 0.0204534260081648 |
| AC069213.2 | ‒ | -3.9531828569857 | 0.0384724111688657 |
| AP000356.2 | ‒ | -3.9511222552957 | 0.0280603656973256 |
| AC023051.1 | ‒ | -3.9200152035844 | 0.0414828260136305 |
| CLSTN2 | calsyntenin 2 | -3.8671399077646 | 0.0420715238656027 |
| AC008581.2 | ‒ | -3.8532508240544 | 0.0318125121477224 |
| KPNA2P1 | karyopherin subunit alpha 2 pseudogene 1 | -3.8427555520414 | 0.0440306296725191 |
| TNFRSF10C | TNF receptor superfamily member 10c | -3.8320078421588 | 0.0439753497998583 |
| AL353708.1 | ‒ | -3.7509962485701 | 0.0402384190778598 |
|  |  |  |  |
|  |  |  |  |
| **ΔHIF1ΔEPAS1_Roxa vs ΔHIF1ΔEPAS1_5%_up** | |  |  |
|  |  |  |  |
| **gene_name** | **gene_description** | **log2FoldChange** | **pvalue** |
| RAB44 | RAB44, member RAS oncogene family | 4.43147383744201 | 0.0114963841488993 |
| TAC4 | tachykinin 4 | 4.29938255809375 | 0.0136549191575972 |
| IRX6 | iroquois homeobox 6 | 4.27643954676251 | 0.0138934300971196 |
| MROH7 | maestro heat like repeat family member 7 | 4.21526253730117 | 0.0338495626905336 |
| ANGPTL4 | angiopoietin like 4 | 4.03481050968723 | 8.7978057190112e-106 |
| AC079922.1 | ‒ | 4.01700823596797 | 0.0290375212828793 |
| AC138207.3 | ‒ | 3.93205967413021 | 0.0127971923169908 |
| AC022973.3 | ‒ | 3.67003050933242 | 0.0226605857692435 |
| CCDC92B | coiled-coil domain containing 92B | 3.59482485457105 | 0.0289835915516324 |
| FILIP1L | filamin A interacting protein 1 like | 3.59258359875987 | 0.0150228861967677 |
| HEXDC-IT1 | HEXDC intronic transcript 1 | 3.57379801879588 | 0.0283933931025906 |
| AC139272.1 | ‒ | 3.55956040713841 | 0.0116073804931782 |
| AC091153.2 | ‒ | 3.47263615474527 | 0.0419881329404442 |
| PRF1 | perforin 1 | 3.44958590785904 | 0.0422929551287605 |
| AC113410.1 | ‒ | 3.38954799504244 | 0.0486557703079607 |
| LINC01297 | long intergenic non-protein coding RNA 1297 | 3.38952780669328 | 0.0489270591555231 |
| AL110115.1 | ‒ | 3.3895225030957 | 0.0489986831912477 |
| RF02039 | ‒ | 3.29589677754106 | 0.0278447926629382 |
| BTF3P7 | basic transcription factor 3 pseudogene 7 | 3.24802877444933 | 0.0274020121604096 |
| LAGE3P1 | L antigen family member 3 pseudogene 1 | 3.18983379718521 | 0.0379555522735144 |
|  |  |  |  |
|  |  |  |  |
| **ΔHIF1ΔEPAS1_Roxa vs ΔHIF1ΔEPAS1_5%_down** | |  |  |
|  |  |  |  |
| **gene_name** | **gene_description** | **log2FoldChange** | **pvalue** |
| TTYH1 | tweety family member 1 | -4.4159484708801 | 0.0086207622653908 |
| AC104695.1 | ‒ | -4.275846128436 | 0.0104336866473935 |
| PGC | progastricsin | -4.1776015872331 | 0.0213086207899371 |
| CR769775.4 | ‒ | -4.0567850197357 | 0.0210786856157868 |
| BX005040.4 | ‒ | -4.0518257952064 | 0.0199828658191368 |
| PSG6 | pregnancy specific beta-1-glycoprotein 6 | -4.0477241794998 | 0.0303406359236037 |
| TOX2 | TOX high mobility group box family member 2 | -3.9180357442009 | 0.0090516702520155 |
| COL20A1 | collagen type XX alpha 1 chain | -3.8196453336853 | 0.0388662412960011 |
| AC008040.1 | ‒ | -3.8077097064386 | 0.0360396855758217 |
| AC007639.1 | ‒ | -3.8064980457902 | 0.0361216733561968 |
| SMARCE1P5 | SWI/SNF related, matrix associated, actin dependent regulator of chromatin, subfamily e, member 1 pseudogene 5 | -3.8050650136367 | 0.0399143601181898 |
| AP000695.3 | ‒ | -3.8028237767676 | 0.0333543551421014 |
| FAM71F1 | family with sequence similarity 71 member F1 | -3.7917496697098 | 0.0371352706123496 |
| LINC00313 | ‒ | -3.7719392771629 | 0.0206392229118594 |
| TBC1D3D | TBC1 domain family member 3D | -3.7684892953517 | 0.0220164344125558 |
| LINC00173 | ‒ | -3.6684646428677 | 0.0059135484461750 |
| KIF25-AS1 | KIF25 antisense RNA 1 | -3.656968249634 | 0.0492041317456691 |
| TMC2 | transmembrane channel like 2 | -3.5252098227442 | 0.0248166128694425 |
| AC007686.1 | ‒ | -3.3198660350859 | 0.0380191760948364 |
| COL8A2 | collagen type VIII alpha 2 chain | -3.3183744561146 | 0.0391185827558056 |

**
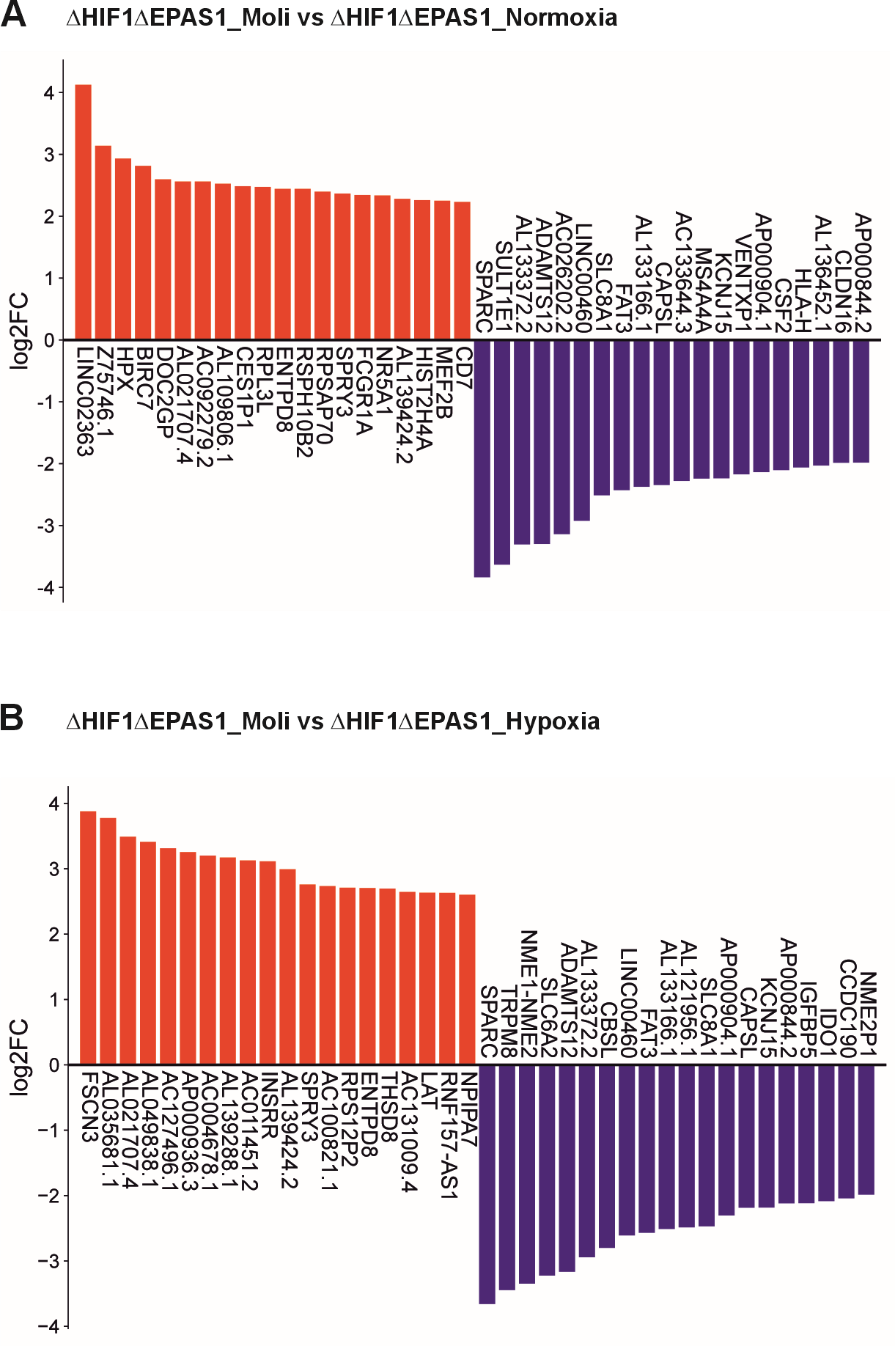
**

**Figure S10. The 20 most significant up- and downregulated genes in ΔHIF1ΔEPAS1 cells treated with molidustat.**

**(A)** Most up- and downregulated genes in ΔHIF1ΔEPAS1 cells upon molidustat (Moli) treatment compared to normoxia. **(B)** Most up- and downregulated genes in ΔHIF1ΔEPAS1 cells upon molidustat (Moli) treatment compared to hypoxic conditions.

**Table S2: List of the 20 most significant up- and downregulated genes in ΔHIF1ΔEPAS1 cells treated with molidustat.**

| **ΔHIF1ΔEPAS1_Moli% vs ΔHIF1ΔEPAS1_16%_up** | |  |  |
| --- | --- | --- | --- |
|  |  |  |  |
| **gene_name** | **gene description** | **log2FoldChange** | **pvalue** |
| LINC02363 | long intergenic non-protein coding RNA 2363 | 4.125191639166 | 0.00111694839126803 |
| RN7SL5P | RNA, 7SL, cytoplasmic 5, pseudogene | 3.83247004621268 | 2.24434576558843e-12 |
| LGI4 | leucine rich repeat LGI family member 4 | 3.39309231109637 | 0.000146535129200782 |
| MTND1P23 | MT-ND1 pseudogene 23 | 3.2187088806798 | 0.00188739351070372 |
| Z75746.1 | zinc finger protein 770 (ZNF770) pseudogene | 3.13848037835054 | 0.00251901795116945 |
| FTH1P10 | ferritin heavy chain 1 pseudogene 10 | 2.99299591511548 | 0.0020820008867955 |
| HPX | hemopexin | 2.93096466163835 | 1.20741462622584e-05 |
| BIRC7 | baculoviral IAP repeat containing 7 | 2.81441480532244 | 0.00375300046973752 |
| ASPDH | aspartate dehydrogenase domain containing | 2.81333130087414 | 1.4597319433785e-07 |
| AC105118.1 | novel transcript | 2.71862813259455 | 0.00804908241493532 |
| DLX5 | distal-less homeobox 5 | 2.63815707790216 | 1.95859006628022e-08 |
| DOC2GP | double C2 domain gamma pseudogene | 2.59668386975338 | 0.000189122459722773 |
| AL021707.4 | novel transcript | 2.56120764252375 | 0.00536148332430468 |
| AC092279.2 | ‒ | 2.56045241726826 | 0.00688952597684355 |
| RTN1 | reticulon 1 | 2.52805086639081 | 5.14334581574025e-09 |
| AL109806.1 | ‒ | 2.52565068951881 | 0.00504066326704339 |
| CES1P1 | carboxylesterase 1 pseudogene 1 | 2.48433117198925 | 0.00748627916156018 |
| AC025165.1 | ‒ | 2.47661838241036 | 8.76339746228076e-07 |
| RPL3L | ribosomal protein L3 like | 2.47217387533416 | 0.00083921254639391 |
| DNM1P35 | dynamin 1 pseudogene 35 | 2.45180760345262 | 0.00487633606619009 |
|  |  |  |  |
|  |  |  |  |
| **ΔHIF1ΔEPAS1_Moli% vs ΔHIF1ΔEPAS1_16%_down** | |  |  |
|  |  |  |  |
| **gene_name** | **gene description** | **log2FoldChange** | **pvalue** |
| SPARC | secreted protein acidic and cysteine rich | -3.8369246857312 | 0.000141175381471456 |
| AL355032.1 | ribosomal protein L26-like 1 (RPL26L1) pseudogene | -3.7609805803694 | 1.45390554295429e-71 |
| SULT1E1 | sulfotransferase family 1E member 1 | -3.6318218643488 | 0.000226978768731904 |
| SLC6A2 | solute carrier family 6 member 2 | -3.4839005071521 | 0.000771267068747036 |
| AC078817.1 | ribosomal protein L26 (RPL26) pseudogene | -3.3666906907108 | 5.69285781168308e-07 |
| AL133372.2 | novel transcript | -3.3042155588911 | 0.000270849241932067 |
| ADAMTS12 | ADAM metallopeptidase with thrombospondin type 1 motif 12 | -3.2966800253899 | 3.07322860387379e-07 |
| AC026202.2 | novel transcript, antisense to ARL8B | -3.1400248994270 | 0.0029449039126687 |
| CEACAM6 | carcinoembryonic antigen related cell adhesion molecule 6 | -3.0392018691646 | 8.92161198667905e-11 |
| LINC00460 | long intergenic non-protein coding RNA 460 | -2.9231351609495 | 0.000285340227467184 |
| AC091804.1 | ezrin (EZR) pseudogene | -2.7862149754643 | 5.129743715994e-07 |
| SERPINB4 | serpin family B member 4 | -2.7770337919236 | 7.84088682202909e-19 |
| KCNU1 | potassium calcium-activated channel subfamily U member 1 | -2.7188025288494 | 3.79517164576709e-05 |
| AC245595.1 | ‒ | -2.5837797619900 | 0.000839945229497557 |
| SLC8A1 | solute carrier family 8 member A1 | -2.5108555561112 | 0.000509263919470933 |
| LINC02432 | long intergenic non-protein coding RNA 2432 | -2.4705489114297 | 0.000682620275337847 |
| FAT3 | FAT atypical cadherin 3 | -2.4290303764695 | 0.00123572570975092 |
| AC005912.1 | ribosomal protein S27 (RPS27) pseudogene | -2.4105031460297 | 1.4982872230834e-135 |
| AL133166.1 | novel transcript | -2.3739838996334 | 0.0174892238426419 |
| LINC01111 | long intergenic non-protein coding RNA 1111 | -2.3586488142204 | 7.13957720685815e-12 |
|  |  |  |  |
|  |  |  |  |
| **ΔHIF1ΔEPAS1_Moli% vs ΔHIF1ΔEPAS1_5%_up** | |  |  |
|  |  |  |  |
| **gene_name** | **gene description** | **log2FoldChange** | **pvalue** |
| FSCN3 | fascin actin-bundling protein 3 | 3.87952780294271 | 0.00283976315212858 |
| AL035681.1 | novel transcript, antisense to L3MBTL2 | 3.7759759604687 | 0.00431176889058696 |
| AL021707.4 | novel transcript | 3.48989915676668 | 0.00268739461748927 |
| AL049838.1 | novel transcript, overlapping C14orf37 | 3.41235716913338 | 0.00272233527603503 |
| CADM2 | cell adhesion molecule 2 | 3.38825940973766 | 0.000235097515792054 |
| AC127496.1 | novel transcript, antisense to RPTOR | 3.31324451391683 | 0.00572491917285015 |
| CA8 | carbonic anhydrase 8 | 3.30615675473237 | 0.000237500627846509 |
| SSC5D | scavenger receptor cysteine rich family member with 5 domains | 3.27725785345986 | 0.00510234583541764 |
| AP000936.3 | ribosomal protein S27 (metallopanstimulin 1) (RPS27) pseudogene | 3.25486469941206 | 0.00524102680183323 |
| AC004678.1 | novel pseudogene | 3.20198082985856 | 0.00687171875566619 |
| AL139288.1 | novel transcript | 3.1726056168871 | 0.00682659559961707 |
| AL136295.6 | novel transcript | 3.15019461480246 | 0.00271481955814129 |
| AC011451.2 | UBX domain protein 2A (UBXN2A) pseudogene | 3.12598039865234 | 0.00904861859961816 |
| INSRR | insulin receptor related receptor | 3.11258506385599 | 0.00911828949676872 |
| AL139424.2 | novel transcript | 2.99296069047 | 0.00715073725152314 |
| AC243772.2 | novel transcript, antisense to FCGR1A | 2.84722920060711 | 6.47611340525519e-07 |
| AC233723.2 | ‒ | 2.80726706196701 | 9.76096338058895e-07 |
| SPRY3 | sprouty RTK signaling antagonist 3 | 2.75821996013914 | 0.00437595603537586 |
| AC100821.1 | ESF1, nucleolar pre-rRNA processing protein, homolog | 2.73362718377227 | 0.00618958536275403 |
| LINC02363 | long intergenic non-protein coding RNA 2363 | 2.72322349520283 | 0.00748352115913129 |
|  |  |  |  |
|  |  |  |  |
| **ΔHIF1ΔEPAS1_Moli% vs ΔHIF1ΔEPAS1_5%_down** | |  |  |
|  |  |  |  |
| **gene_name** | **gene description** | **log2FoldChange** | **pvalue** |
| AC078817.1 | ribosomal protein L26 (RPL26) pseudogene | -3.7210911618622 | 1.06871796823022e-08 |
| SPARC | secreted protein acidic and cysteine rich | -3.6574743572470 | 0.000784690384454086 |
| AL355032.1 | ribosomal protein L26-like 1 (RPL26L1) pseudogene | -3.6121404543259 | 2.49849978020154e-61 |
| TRPM8 | transient receptor potential cation channel subfamily M member 8 | -3.4462676291313 | 0.00329710871686214 |
| NME1-NME2 | NME1-NME2 readthrough | -3.3477050857833 | 0.00100043456624711 |
| SLC6A2 | solute carrier family 6 member 2 [ | -3.222618485797 | 0.00396796302031868 |
| ADAMTS12 | ADAM metallopeptidase with thrombospondin type 1 motif 12 | -3.1653874889211 | 9.507643083536e-07 |
| CEACAM6 | carcinoembryonic antigen related cell adhesion molecule 6 | -3.1385768718485 | 2.03305997951004e-09 |
| AL133372.2 | novel transcript | -2.9437133556175 | 0.00123086887778334 |
| CBSL | cystathionine-beta-synthase like | -2.8000447132517 | 0.00170769208732752 |
| AC091804.1 | ezrin (EZR) pseudogene | -2.6908695393494 | 1.97561484648598e-06 |
| LINC00460 | long intergenic non-protein coding RNA 460 | -2.6080415797804 | 0.00342339018576234 |
| FAT3 | FAT atypical cadherin 3 | -2.5681300927589 | 0.000208419855603779 |
| SERPINB4 | serpin family B member 4 | -2.5238995154585 | 9.15004517448924e-15 |
| AL133166.1 | novel transcript | -2.5101699939773 | 0.00795073988591321 |
| AL121956.1 | uncharacterized LOC101929297 | -2.4853259103739 | 0.00632766798632879 |
| SLC8A1 | solute carrier family 8 member A1 | -2.4694516580972 | 0.00129768678253616 |
| KCNU1 | potassium calcium-activated channel subfamily U member 1 | -2.3999706844395 | 0.000414273880826074 |
| TCN1 | transcobalamin 1 | -2.3996811640443 | 5.31286561989932e-11 |
| LINC01111 | long intergenic non-protein coding RNA 1111 | -2.3567798376163 | 2.20542340901587e-12 |

**Table S3: Overlap between our Top‑20 lists and GSE77789 (Egln1/PHD2 KO) Top‑250 (GEO2R)**

| **Gene** | **Egln1/PHD2 KO (GSE77789)** | **Our Top‑20 direction (context)** | **Concordance** |
| --- | --- | --- | --- |
| **IGFBP3** | Up | **Up**  (multiple contrasts incl. roxadustat in control cells) | **Concordant** |
| **HMCN1** | Up | **Up**  (ΔHIF1ΔEPAS1_16% vs Ctl_16%) | **Concordant** |
| **CDH11** | Up | **Down**  (Ctl_Roxa vs Ctl_16%; Ctl_Roxa vs Ctl_5%; ΔHIF1ΔEPAS1_Roxa vs ΔHIF1ΔEPAS1_5%) | **Discordant** |
| **PCDH18** | Up | **Down**  (Ctl_Roxa vs Ctl_5%) | **Discordant** |
| **TTC5** | Up | **Down**  (Ctl_5% vs Ctl_16%) | **Discordant** |
| **PHLDA3** | Down | **Up**  (ΔHIF1ΔEPAS1_Roxa vs ΔHIF1ΔEPAS1_16%) | **Discordant** |
| **STC1** | Down | **Up**  (Ctl_5% vs Ctl_16%; Ctl_Moli vs Ctl_16%; Ctl_Moli vs Ctl_5%) | **Discordant** |

**Table S4: Nucleotide sequences used for CRISPR-Cas9 gene editing.**

| **Gene** | **Nucleotide sequence (5' to 3')** |
| --- | --- |
| *HIF1A* (guide) | CCATCAGCTATTTGCGTGTG |
| *EPAS1* (guide) | CAAGGCCTCCATCATGCGAC |
| *Egln1* (guide) | CACCGCGGGCAGCAGATCGGCGATG |
| *Egln2* (guide) | CACCGAGGTTCCCGCGAATGTTGCG |
| *Egln3* (guide) | CACCGCGTGCGCCGGCGTCTCCAAG |
| **Genotyping/ Sequencing** | **Nucleotide sequence (5' to 3')** |
| *HIF1A* | Forward: GAAAAGTCTCGAGATGCAGCCA |
|  | Reverse: GGGAAAAGCCAGTATCTTATTCCTG |
| *EPAS1* | Forward: CCCATGTGAAGCCCTGTTCT |
|  | Reverse: CCCATGTTCTTCCCTGGTCC |
| *Egln1* | Forward:ACTTACTTTCGTTCGGCCGT  Reverse:TCCGAGGATCCTCCGCTTAG |
| *Egln2* | Forward:CCAGTAGGCTTCCTCCCGTA  Reverse:AGCGACGATGCAAGTGGAAA |
| *Egln3* | Forward:TGGATCTGGAGAAGATCGCC  Reverse:ATCCACGTGATCTGGTCGC |
| *CBFA2T3*  (off-target *HIF1A*) | Forward: GAGGAGCTCAACCACTGGG |
|  | Reverse: CTCGGACAAGGTCTGGCTC |
| *ALDH1L1*  (off-target *EPAS1*) | Forward: TGATCTCTCAGTGTCACAGCCA |
|  | Reverse: GCCCCTTCACACCCTTATCC |

**Table S5: List of antibodies used in the study.**

| **Primary Antibodies** | **Origin** | **Clonality** | **Dilution**  **factor** | **Company** | **Catalog nr.** |
| --- | --- | --- | --- | --- | --- |
| HIF-1α | Mouse | monoclonal | 1:1.000 | BD Bioscience | #610959 |
| HIF-2α (D9E3) | Rabbit | monoclonal | 1:1.000 | Cell signaling | #7096 |
| CA IX (H-11) | Mouse | monoclonal | 1:1.000 | Santa Cruz | #sc-365900 |
| BNIP3 (D7U1T) | Rabbit | monoclonal | 1:1.000 | Cell signaling | #44060 |
| PHD1 | Rabbit | monoclonal | 1:1000 | Cell signaling | #33985 |
| PHD2 | Rabbit | polyclonal | 1:1000 | Cell signaling | #3293 |
| PHD3 | Rabbit | monoclonal | 1:500 | Abcam | #ab184714 |
| α-Tubulin  (clone B-5-1-2) | Mouse | monoclonal | 1:10.000 | Sigma-Aldich | #T5168 |
| **Secondary Antibodies** | **Origin** |  | **Dilution**  **factor** | **Company** | **Catalog nr.** |
| Mouse-HRP | Goat |  | 1:5.000 | Bio-Rad | #1706516 |
| Rabbit-HRP | Goat |  | 1:5.000 | Bio-Rad | #1706515 |
